# Sensitivity and specificity of human point-of-care circulating cathodic antigen (POC-CCA) test in African livestock for rapid diagnosis of schistosomiasis: A Bayesian latent class analysis

**DOI:** 10.1371/journal.pntd.0010739

**Published:** 2023-05-22

**Authors:** Beatriz Calvo-Urbano, Elsa Léger, Isobel Gabain, Claudia J. De Dood, Nicolas D. Diouf, Anna Borlase, James W. Rudge, Paul L. A. M. Corstjens, Mariama Sène, Govert J. Van Dam, Martin Walker, Joanne P. Webster

**Affiliations:** 1 Royal Veterinary College, Department of Pathobiology and Population Sciences, University of London, Hatfield, United Kingdom; 2 London Centre for Neglected Tropical Disease Research, School of Public Health, Department of Infectious Disease Epidemiology, Imperial College Faculty of Medicine, London, United Kingdom; 3 Leiden University Medical Centre, Leiden, The Netherlands; 4 Unité de Formation et de Recherche des Sciences Agronomiques, d’Aquaculture et de Technologies Alimentaires, Université Gaston Berger, Saint Louis, Senegal; 5 Department of Biology, University of Oxford, Oxford, United Kingdom; 6 Communicable Diseases Policy Research Group, Department of Global Health and Development, London School of Hygiene and Tropical Medicine, London, United Kingdom; 7 Faculty of Public Health, Mahidol University, Bangkok, Thailand; The University of Sydney School of Veterinary Science, AUSTRALIA

## Abstract

Schistosomiasis is a major neglected tropical disease (NTD) affecting both humans and animals. The morbidity and mortality inflicted upon livestock in the Afrotropical region has been largely overlooked, in part due to a lack of validated sensitive and specific tests, which do not require specialist training or equipment to deliver and interpret. As stressed within the recent WHO NTD 2021–2030 Roadmap and Revised Guideline for schistosomiasis, inexpensive, non-invasive, and sensitive diagnostic tests for livestock-use would also facilitate both prevalence mapping and appropriate intervention programmes. The aim of this study was to assess the sensitivity and specificity of the currently available point-of-care circulating cathodic antigen test (POC-CCA), designed for *Schistosoma mansoni* detection in humans, for the detection of intestinal livestock schistosomiasis caused by *Schistosoma bovis* and *Schistosoma curassoni*. POC-CCA, together with the circulating anodic antigen (CAA) test, miracidial hatching technique (MHT), Kato-Katz (KK) and organ and mesentery inspection (for animals from abattoirs only), were applied to samples collected from 195 animals (56 cattle and 139 small ruminants (goats and sheep) from abattoirs and living populations) from Senegal. POC-CCA sensitivity was greater in the *S*. *curassoni*-dominated Barkedji livestock, both for cattle (median 81%; 95% credible interval (CrI): 55%-98%) and small ruminants (49%; CrI: 29%-87%), than in the *S*. *bovis*-dominated Richard Toll ruminants (cattle: 62%; CrI: 41%-84%; small ruminants: 12%, CrI: 1%-37%). Overall, sensitivity was greater in cattle than in small ruminants. Small ruminants POC-CCA specificity was similar in both locations (91%; CrI: 77%-99%), whilst cattle POC-CCA specificity could not be assessed owing to the low number of uninfected cattle surveyed. Our results indicate that, whilst the current POC-CCA does represent a potential diagnostic tool for cattle and possibly for predominantly *S*. *curassoni*-infected livestock, future work is needed to develop parasite- and/or livestock-specific affordable and field-applicable diagnostic tests to enable determination of the true extent of livestock schistosomiasis.

## Author summary

Schistosomiasis is a neglected tropical and zoonotic disease, infecting over 230 million people and millions of animals worldwide. The potential contribution of livestock schistosomiasis to disease transmission in human populations has implications for the design of effective disease management and elimination programmes. However, our understanding of the true prevalence and impact of animal schistosomiasis is severely limited, in part due to a lack of accessible and accurate diagnostic tools. This need for sensitive and specific tools for animal schistosomiasis diagnosis has been recognised in the most recent WHO Guideline and NTD roadmap. As a point-of-care circulating cathodic antigen (POC-CCA) diagnostic test is now available to assess intestinal schistosomiasis caused by *Schistosoma mansoni* in humans, we hypothesised that POC-CCA could be used to detect livestock intestinal schistosomiasis caused by *Schistosoma bovis* and *Schistosoma curassoni*. The aim of this study was to evaluate the sensitivity and specificity of POC-CCA for the detection of intestinal livestock schistosomiasis in Senegal. POC-CCA sensitivity varied by ruminant group and by parasite species/location, while POC-CCA specificity in small ruminants, at least, did not vary across populations. We conclude that, whilst the currently-available POC-CCA does represent a potential diagnostic tool for schistosomiasis in cattle, the factors determining test performance warrant further investigation and further livestock-specific assays would be ideal.

## Introduction

The development and application of sensitive and specific diagnostic techniques for detection of infectious diseases is vital for the monitoring and evaluation of all treatment programs in endemic areas, especially whenever considering elimination and/or drug-resistance pharmacovigilance. Within this, point-of-care diagnostic testing is particularly needed wherever there is a necessity for a fast diagnostic outcome that is independent from sophisticated, time-consuming, labour-intense and/or expensive laboratory procedures [1]. This need may be most exemplified for the neglected tropical and zoonotic diseases (NTDs/NZDs), as clearly stressed within the recent World Health Organization’s (WHO) NTD Roadmap for 2021–2030 [2] and revised WHO Guideline for the control and elimination of human schistosomiasis, most notably that of recommendation 6 [3, 4].

Schistosomiasis is one of the major debilitating NTDs/NZDs, caused by snail-borne dioecious *Schistosoma* spp. blood-flukes. Approximately 90% of the 230 million people infected worldwide live in the Afrotropical region [2]. Animal schistosomiasis is also of major veterinary and socio-economic importance, causing significant mortality and morbidity to livestock, as well as reduced productivity for their owners, although the contribution and consequences of this within the Afrotropical region are only just beginning to be realised [2, 5–8]. The main *Schistosoma* species found amongst livestock in Africa are the intestinal *Schistosoma bovis* and *Schistosoma curassoni* in West Africa and *Schistosoma mattheei* in East Africa. These species are phylogenetically close to the human urogenital parasite *Schistosoma haematobium* and have been found to regularly form viable hybrids within humans, both across Africa [8–13], and even within its recent expanse to Europe [14, 15]. Outside of Asia, the contribution of zoonotic schistosomes to human schistosomiasis cases have been largely ignored, despite prevalence levels in humans often remaining unacceptably high following high coverage mass drug administration (MDA) programmes across much of West Africa in particular [8, 16, 17]. Furthermore, recent work combining epidemiological, molecular and mathematical modelling work has demonstrated that the relative role of zoonotic transmission from livestock in Africa is likely to increase as disease control efforts move towards elimination [18].

The recently launched WHO NTD 2021–2030 Roadmap and Guideline for the control and elimination of human schistosomiasis therefore poses the question of anti-schistosomiasis treatment of livestock across Africa in order to achieve the new targets of Elimination as a Public Health Problem (EPHP) in all 78 currently-endemic counties and Interruption of Transmission (IoT) in selected African regions by 2030 [2, 3]. Widespread, indiscriminate use of anthelmintics in general amongst livestock have increased drug-resistance [19, 20]. This is particularly pertinent for schistosomiasis as there is currently only one efficacious drug, praziquantel (PZQ), available for both humans and livestock. Recent work has highlighted examples of use and misuse of PZQ in livestock, including, but not exclusive to, PZQ tablets donated free via the WHO MDA programmes for school-aged children being used instead for infected livestock, with little knowledge of application or dosage requirements [5, 7]. Furthermore, recent surveys and socio-economic analyses have revealed highly important, if also often overlooked, animal welfare, productivity, and financial impacts of animal schistosomiasis, which further jeopardize livelihoods, food security and nutrition amongst neglected communities [5]. Indeed the financial costs incurred to subsistence farmers of infected animals was projected to be significantly greater than would be those of a theoretical test and treat programme [5].

To promote sustainable livestock schistosomiasis prevention and treatment in the Afrotropical region, there is therefore a need to both easily and inexpensively diagnose animal schistosomiasis, and subsequently effectively treat only infected individuals and/or herds, such as through a targeted test-and-treat (TnT) or *T3*: *Test*, *Treat*, *Track* design for livestock schistosomiasis in the Afrotropical region, which anchors the key recent WHO policy recommendations on diagnostic testing, treatment and surveillance in general.

There are, however, currently limited diagnostic techniques that can detect schistosomiasis in livestock with sufficient levels of sensitivity and specificity, as well as logistical ease, that can fully inform disease management decisions [21]. Of those available, a recent systematic review (although data analysed were exclusive to *Schistosoma mansoni* and *Schistosoma japonicum* infections) has recommended the formalin-ethyl acetate sedimentation-digestion with quantitative polymerase chain reaction, as the most promising field-applicable techniques in non-human animal hosts [22], although both are time consuming and/or expensive. A recent extensive field survey of both abattoir and live-sampled livestock within Senegal found some utility with both the Kato-Katz technique (KK) and miracidial hatching technique (MHT), but the latter was again labour-intensive and showed significant differences in sensitivities by host and/or parasite species [8].

Tests for antigens, rather than antibodies, are preferred in endemic areas due to the lag in clearance of parasite-specific antibodies after infection subsides [23] and therefore immunochromatographic circulating cathodic antigen (CCA) and circulating anodic antigen (CAA) tests for worm antigens in urine (or serum) were developed specifically for current *Schistosoma* spp. infections amongst humans [24]. Whilst a high sensitivity laboratory-based lateral flow (LF) test platform utilizing luminescent up-converting reporter particles (UCP) comprises assay formats for quantitative detection of circulating cathodic and anodic antigen detection in urine (respectively, UCCA and UCAA assays) is available [25, 26], rapid point-of-care testing with visual detection is currently only available for the CCA (POC-CCA, Rapid Medical Diagnostics, Pretoria, South Africa) [27, 28]. Given its utility and field-friendly application, this semi-quantitative (negative, trace, “single positive” +, “double positive” ++ or “triple positive” +++) POC-CCA is now recommended by the WHO for mapping human *S*. *mansoni* prevalence in endemic areas [29, 30]. The application for detection of human urogenital *S*. *haematobium* infections with POC-CCA is less efficient i.e. to find the lower trematode burden infections [31–34].

The POC-CCA has been used to assess *S*. *mansoni* infection in one non-human-primate study [35]). Given that *S*. *bovis* and *S*. *curassoni* (as well as *S*. *mattheei*) are intestinal schistosomes of livestock, and taking into account our recent social surveys which showed a demand amongst livestock owners within Western Africa [5], we predicted that the POC-CCA, when used in partnership with local veterinary technicians, could be a cost-effective tool for intestinal livestock schistosomiasis control. The aim of this study was therefore to evaluate the sensitivity and specificity of the currently available, human-focused, POC-CCA for the detection of intestinal livestock schistosomiasis caused by *S*. *bovis*, *S*. *curassoni* and their hybrids in Senegal, by host species, in relation to traditional and novel alternative parasitological and immunological diagnostic methods currently available, employing Bayesian latent class models.

## Methods

### Ethics approval and consent to participate

For all primary data collection activities, the researchers first explained to the livestock owners and, for live-sampling collections, village leaders what the study was about, how the data collection would work and the rights of the participants. Following that, written consent was obtained from each participant. Ethical approval was sought and granted by: i) the Clinical Research and Ethical Review Board at the Royal Veterinary College; approval number URN 2019 1899–3; and (ii) the Comité National d’Ethique pour la Recherche en Santé (Dakar, Senegal) approval number SEN15/68 and SEN 19/68. Urine samples were imported from Senegal to the RVC in the UK with the import licence ITIMP19.0161 and from the UK to LUMC in the Netherlands with the import licence VGM_IN17-1416-GvW.

### Study sites and diagnostic methods

The study was conducted in Senegal, West Africa. Cross-sectional livestock parasitological surveys were conducted from May to August 2016 and from October 2017 to January 2018 in two areas located in the north of the country; the town of Richard Toll, in the Senegal River Basin, and villages around the Lac de Guiers (area hereafter referred to as Richard Toll), and the town of Linguere and villages around Barkedji along the Vallée du Ferlo (area hereafter referred to as Barkedji). Following the construction of the Diama Dam in 1986, situated on the Senegal River over 100 km downstream from Richard Toll, the environment surrounding this location has undergone important permanent alterations. Desalination, creation of irrigation canals and of permanent freshwater bodies have facilitated the expansion of *Schistosoma* snail intermediate host and human-livestock water contact points throughout the year, supporting the co-occurrence and interspecific interactions between *S*. *haematobium*, *S*. *mansoni*, and other *Schistosoma* spp. of veterinary importance [36–38]. The main livestock schistosome species circulating in Richard Toll is *S*. *bovis* and cattle is the most affected host species. The Barkedji area presents temporary water sources that disappear during the dry season, leading to important seasonal migration of livestock-keeping communities, and annual interruption in schistosomiasis transmission. Small ruminants are the most important livestock host species, infected by *S*. *curassoni*. However, both schistosome species and hybrids are present in the two areas and all livestock species can be found infected to a lesser extent. Full details on the infection prevalence amongst definitive and intermediate hosts, together with the habitat of two areas, can be found in [8].

Animal sampling was part of a larger survey conducted in these two areas [8]. All animals (cattle, sheep, and goats) routinely slaughtered as part of the normal work of the abattoirs and available for inspection at the time of the surveys were examined post-mortem. Living animals were randomly selected in each village with the initial randomisation carried out at the unit level (owner in this case) and a maximum of five animals of each species (cattle, sheep and goats) then randomly sampled from each selected owner (fewer if the owner had less than five animals). Randomisation was carried out using random number generators. The mesenteric vessels of slaughtered animals were visually inspected for *Schistosoma* spp. adults (single males, single females, and paired worms) and those found were stored in RNA-later for molecular analysis (see Sections 1 and 2 in S1 Appendix). Faecal, urine, lung, and liver samples were collected post-mortem and were examined for infection using the miracidial hatching technique (MHT) (see Section 1 in S1 Appendix). Free-swimming miracidia were individually pipetted onto Whatman FTA cards (GE Healthcare Life Sciences, UK) for deoxyribonucleic acid (DNA) storage and subsequent genotyping (see Section 2 in S1 Appendix). Only faecal and urine samples were obtained from live animals. Faecal samples were assessed for the presence of *Schistosoma* spp. eggs via two Kato-Katz (KK) slides and MHT (see Sections 1 and 2 in S1 Appendix). Animals for which a sufficient volume of urine (≥15 mL) was collected were tested on-site for schistosomiasis with a single POC-CCA cassette (Rapid Medical Diagnostics, Pretoria, South Africa) and for haematuria with a single Hemastix strip (Siemens Healthcare Diagnostics, Surrey, UK) (see Section 1 in S1 Appendix). The remaining urine was frozen and transported to Leiden Medical University Centre (LUMC) in the Netherlands for application with the up-converting phosphor-lateral flow (UCP-LF) based assays formats (UCCA and UCAA) for the detection and quantitation of circulating cathodic and anodic antigen in urine (see Section 1 in S1 Appendix) [26]. Definitions of positive results are described in Section 1, Table A in S1 Appendix.

### Statistical analyses

#### Association between haematuria and POC-CCA results

When extracting urine from abattoir animals, some samples became contaminated with blood. As false-positive POC-CCA tests in humans have been associated with haematuria [39], it was of interest to assess whether POC-CCA results were affected by the blood in urine. Hemastix results were dichotomised as negative (score 0) or positive (scores 1 and 2) and by means of logistic regression we tested the hypothesis that POC-CCA results were independent of blood-contamination status.

#### Composite reference standard and Bayesian latent class model specification

Due to the absence of a gold standard for the diagnosis of livestock schistosomiasis against which we could evaluate the performance of POC-CCA, we derived a composite reference standard (CRS) that was based on three additional diagnostic methods, namely MHT, KK and UCAA. CRS is based on a combination of tests with moderate sensitivity and high specificity [40]. CRS results were considered positive if animals tested positive for either UCAA, MHT or KK. CRS results were assumed to be negative if animals tested negative for all three tests.

We then developed a Bayesian latent class model (BLCM) to assess the sensitivity and specificity of POC-CCA for the detection of active schistosomiasis in both large (cattle) and small (sheep and goats) ruminants [41–43] that took account of the imperfect reference diagnostic tests employed. Two latent (i.e. non-observed) classes were assumed, corresponding to either an infected or non-infected status, and these classes were related to the outcomes of POC-CCA, CRS and UCCA by means of a multinomial distribution [41, 44, 45]. We developed a two-test model and a three-test model (see Section 3, Table B in S1 Appendix). The diagnostic tests included in the two-test model were POC-CCA and CRS. The three-test model made use of the UCCA results and comprised POC-CCA, CRS and UCCA outcomes, albeit at the expense of lower sample sizes in each combination of diagnostic tests.

#### Accuracy model assumptions

Latent class models (LCM), as proposed by Hui and Walter [42], involve three assumptions. Firstly, more than one population needs to be assessed, each with distinct disease prevalence. Secondly, diagnostic test accuracy must remain constant across populations. Thirdly, the accuracy of the tests must be conditionally independent, so that the sensitivity or specificity of one test is independent of the results of a second test [41, 44, 45]. In the present study we were planning to model abattoir and live animals as two distinct populations. However, as schistosomiasis observed prevalence in these two populations were similar (see Section 4, Table D in S1 Appendix), it was likely that the assumption of different prevalence levels amongst populations was not satisfied. Hence, abattoir and live data were combined and a one population approach was adopted for each site and host species group [43]. However, and in order to assess whether test accuracy differed in these two populations, independent abattoir and live estimates were subsequently derived and compared. To help overcome potential identifiability problems that a one population approach could entail and taking into account the assumed high specificity and medium sensitivity of CRS, we employed moderately informative priors for the CRS (see Section 3, Table C in S1 Appendix). Equally, prevalence priors were moderately informative and their values were based on results from a study carried out in the same area [8]. The impact that these moderately informative priors had on the accuracy estimates (sensitivity/specificity) was assessed through a sensitivity analysis (see Section 6 in S1 Appendix).

Conditional independence of POC-CCA and CRS in the two-test model was assessed in two ways. Firstly, the Deviance Information Criterion (DIC) [46] of model variants that included (dependence) and excluded (independence) covariance terms were estimated and compared. Models with lower DIC were preferred over models with higher DIC. Secondly, 95% credible interval (CrI) of covariance parameters were assessed [45], with CrIs intersecting zero indicative of conditional independence. Three-test models included dependence terms for POC-CCA and UCCA only as the two tests measure the same antigen (CCA) and their outcomes may not be independent of each other. Furthermore, the covariances between POC-CCA and CRS had been found not to be relevant (see results section “POC-CCA accuracy”).

#### Selection of priors and priors’ sensitivity analyses

The beta prior distributions (Section 3, Table C in S1 Appendix) were parameterized by specifying the mode and the minimum/maximum accuracy value that was believed to be true for each variable, with 95% certainty. Priors for UCAA and UCCA sensitivity and specificity were established based on expert knowledge and published records of high performance [47–50]. Priors for POC-CCA accuracy were non-informative beta (1,1) distributions. Prevalence priors were based on post-mortem examination of abattoir specimens from a previous study [8], where 81% and 82% of cattle were found to be infected in Barkedji and Richard Toll, respectively, and 26% and 16% of small ruminants were found to be infected in Barkedji and Richard Toll, respectively. As the proportion of infected animals were similar at the two sites, only one prior distribution per ruminant group was defined (Section 3, Table C in S1 Appendix). The sensitivity of the results to the prior parameterisations was undertaken by re-estimating POC-CCA accuracy assuming more diffuse priors for prevalence and CRS accuracy (see Section 6, Table F in S1 Appendix).

#### Accuracy comparisons between sites

In order to assess whether test accuracy differed between sites, differences in the posterior distributions of accuracy between sites were calculated and the probability that the accuracy in one location was greater than in the other was calculated (the Bayesian p-value) by means of the JAGS “step” function [51, 52]. When accuracy did not differ between sites, combined accuracy values were calculated.

#### Direct diagnostic and POC-CCA accuracy

Direct diagnostic of schistosomiasis in abattoir animals enabled us to determine whether animals were infected with adult trematodes and miracidia. Two-test independence models were applied to these data and POC-CCA results. This enabled us to estimate POC-CCA sensitivity in abattoir animals and to assess whether CRS-derived and direct diagnostic-derived sensitivities differed. Sensitivity and specificity for direct diagnostic were considered to be 99%.

#### Software

Statistical analyses were carried out in R [53] version 4.0.5 (see Sections 3 and 8 in S1 Appendix).

## Results

### Descriptive statistics

Abattoir surveys were carried out on 89 animals routinely slaughtered, whilst live animal samples were obtained from 106 cattle, sheep and goats from communities within the study area. Given the low number of goats and sheep that were infected in the present study, their data were grouped and analysed as “small ruminants” (see Table 1). A total of 195 animals were surveyed (56 cattle and 139 small ruminants). POC-CCA results were missing from four cattle and one sheep. The distributions of samples across sites and sources of animals (live or abattoir) are shown in Table 1. Barkedji livestock was primarily infected by *S*. *curassoni*, whilst animals from Richard Toll shed mainly *S*. *bovis* eggs (see Section 5, Table E in S1 Appendix).

**Table 1:** Number of live and abattoir livestock animals surveyed in Barkedji and Richard Toll.

Site		Total	Bovine	Small ruminant (goat + sheep)
Barkedji	Abattoir	35	13	22 (19+3)
	Live	76	8	68 (11+57)
	Total	111	21	90 (30+60)
Richard Toll	Abattoir	54	28	26 (25+1)
	Live	30	7	23 (2+21)
	Total	84	35	49 (27 +22)
Both sites	Abattoir	89	41	48 (44 +4)
	Live	106	15	91 (13 + 78)
	Total	195	56	139 (57 + 82)

### Association between haematuria and POC-CCA results

Logistic regression results indicated that abattoir POC-CCA results did not depend on Hemastix outcomes, either in cattle (coefficient z value: -0.36, p-value: 0.719) nor in small ruminants (coefficient z value: 0.773, p-value: 0.444). Consequently, in all subsequent analyses, no distinction was made between blood-positive or negative samples.

### Cross-tabulated results

The cross-tabulated results for CRS and POC-CCA are shown in Table 2. The number of small ruminants’ discordant pairs (POC-CCA -, CRS +) was relatively large, suggesting that POC-CCA sensitivity in small ruminants might be low. Cattle were CRS negative in 8 out of 56 animals, showing that most animals were infected, which limited our ability to estimate POC-CCA specificity in cattle. The number of (+, +) concordant pairs was greater in cattle than in small ruminants and the number of (-,-) concordant pairs were greater in small ruminants than in cattle, suggesting that the test behaved differently in each ruminant group.

**Table 2:** Cross-tabulated results for POC-CCA and CRS, by site and ruminant group.

		Number of concordant and discordant pairs (POC-CCA, CRS)*			
Site	Ruminant	(+, +)	(+, -)	(-, +)	(-, -)
Barkedji	Cattle **	13	1	3	1
	Small ruminants **	19	6	29	35
Richard Toll	Cattle **	17	2	11	4
	Small ruminants	3	2	26	18

* POC-CCA = point-of-care cathodic circulation antigen, CRS = composite reference standard

** POC-CCA readings were missing for 3 cattle and 1 small ruminant in Barkedji, and for 1 cattle in Richard Toll.

### POC-CCA accuracy

#### Conditional dependence between tests and selection of the best fitting models

BLCM results from the two-test dependence and two-test independence models, by site and ruminant group (Table 3), indicate that POC-CCA and CRS are conditionally independent as: 1) the 95% CrIs of the covariance terms in the two-test model intersected zero; and 2) independence models had lower DIC values than the dependence ones. Results from the three-test model indicate that POC-CCA and UCCA sensitivities were conditionally dependent (lower 95% CrI limit was greater than zero) in all cases with the exception of the small ruminants from Richard Toll (where the CrI contained zero) (Tables 3 and 4). By contrast, POC-CCA and UCCA specificities were conditionally independent, their 95% CrI intersecting zero (Tables 3 and 4). Given that the DIC of the two-test independence models were the lowest, we adopted these as the best fitting models and the following conclusions on POC-CCA accuracy were derived from them.

**Table 3:** Cattle Bayesian latent class models results from two-test and three-test models showing parameter medians (95% CrI) for POC-CCA accuracy.

Site	Variable	Two-Test Independence	Two-Test Dependence	Three-Test
B	Sensitivity (%)	81 (55, 98)	77 (52, 97)	72 (48, 94)
	Specificity (%)	55 (5, 98)	51 (3, 97)	61 (13, 96)
	Se cov	n.a.	0.04 (-0.04, 0.14)	0.13 (0.01, 0.22)
	Sp cov	n.a.	0 (-0.12, 0.12)	-0.03 (-0.3, 0.14)
	DIC	47.96	49.38	66.98
R	Sensitivity (%)	62 (41, 84)	58 (38, 82)	60 (41, 80)
	Specificity (%)	70 (16, 98)	63 (10, 98)	82 (40, 99)
	Se cov	n.a.	0.03 (-0.06, 0.11)	0.19 (0.1, 0.23)
	Sp cov	n.a.	0.01 (-0.1, 0.14)	0.04 (-0.09, 0.19)
	DIC	54.27	54.87	76.05
B & R	Sensitivity (%)	--	66 (49, 86)	64 (48, 80)
	Specificity (%)	--	61 (8, 98)	71 (31, 97)
	Se cov	n.a.	0.03 (-0.04, 0.1)	0.19 (0.12, 0.23)
	Sp cov	n.a.	0.01 (-0.11, 0.14)	0.05 (-0.06, 0.17)
	DIC	54.40	55.23	77.89
	Pr(Se B > Se R)	0.88	0.86	0.78
	Pr(Sp B > Sp R)	0.36	0.38	0.26

Se = sensitivity; Sp = specificity; cov = covariance; DIC = Deviance information criterion; B = Barkedji; R = Richard Toll; n.a. = not applicable; Pr = probability

**Table 4:** Small ruminants Bayesian latent class models results from two-test and three-test models showing parameter medians (95% CrI) for POC-CCA accuracy.

Site	Variable	Two-Test Independence	Two-Test Dependence	Three-Tests
B	Sensitivity (%)	49 (29, 87)	42 (7, 85)	41 (25, 66)
	Specificity (%)	91 (73, 99)	85 (58, 99)	91 (75, 99)
	Se cov	n.a.	0 (-0.1, 0.1)	0.09 (0, 0.16)
	Sp cov	n.a.	0.03 (-0.03, 0.17)	0.02 (-0.03, 0.12)
	DIC	55.92	55.94	107.55
R	Sensitivity (%)	12 (1, 37)	13 (1, 40)	26 (8, 68)
	Specificity (%)	88 (65, 99)	89 (65, 99)	93 (74, 100)
	Se cov	n.a.	-0.02 (-0.13, 0.03)	0.12 (-0.01, 0.21)
	Sp cov	n.a.	0.02 (-0.03, 0.11)	0.01 (-0.05, 0.1)
	DIC	46.53	55.92	92.92
B & R	Sensitivity (%)	n.a.	30 (3, 69)	34 (19, 65)
	Specificity (%)	91 (77, 99)	86 (61, 99)	92 (80, 99)
	Se cov	n.a.	-0.01 (-0.1, 0.07)	0.12 (0.04, 0.19)
	Sp cov	n.a.	0.03 (-0.02, 0.16)	0.02 (-0.01, 0.09)
	DIC	54.01	54.51	106.18
	Pr(Se B > Se R)	0.99	0.91	0.80
	Pr(Sp B > Sp R)	0.60	0.41	0.45

Se = sensitivity; Sp = specificity; cov = covariance; DIC = Deviance information criterion; B = Barkedji; R = Richard Toll; n.a. = not applicable; Pr = probability

#### POC-CCA accuracy in cattle

POC-CCA sensitivity in Barkedji was 81% (95% CrI: 55% to 98%) and in Richard Toll it was 62% (95% CrI: 41% to 84%). As the probability that the sensitivity in Barkedji was greater than in Richard Toll was high (0.88, see Pr in Table 3), a combined sensitivity value for both sites was not calculated.

This study was not able to estimate POC-CCA specificity in cattle with precision due to the low number of animals that were not infected. This is shown by the wide credible intervals obtained in the analyses (see Table 3).

#### POC-CCA accuracy in small ruminants

POC-CCA sensitivity in Barkedji was 49% (95% CrI: 29% to 87%) and in Richard Toll it was 12% (95% CrI: 1% to 37%) (see Table 4). The probability that the sensitivity in Barkedji was greater than in Richard Toll was 0.99 (see Pr in Table 4). As this difference was large, overall sensitivity across sites was not estimated.

POC-CCA specificity in Barkedji was 91% (95% CrI: 73% to 99%) and in Richard Toll it was 88% (95% CrI: 65% to 99%). The probability that the specificity in Barkedji was greater than in Richard Toll was 0.60. The overall specificity was 91% (95% CrI: 77% to 99%).

#### Direct diagnostic and POC-CCA accuracy

Table 5 shows that the sensitivity and specificity results derived from BLCM that included POC-CCA and CRS (live and abattoir animals) were comparable to those obtained from BLCM that comprised POC-CCA and direct diagnostic (abattoir animals only). The main difference between the two approaches being that sensitivity in small ruminants in Richard Toll could not be assessed with precision when using direct diagnostic.

**Table 5:** Two-test independence models results showing POC-CCA sensitivity and specificity medians (95% CrI) derived from models including POC-CCA and CRS or POC-CCA and direct diagnostic.

Parameter	Ruminant	Site	POC-CCA & CRS	POC-CCA & direct diagnostic	n*
Sensitivity	Cattle	Barkedji	81% (55, 98)	77% (48, 94)	12
		Richard Toll	62% (40, 84)	64% (43, 82)	27
	Small ruminants	Barkedji	49% (29, 87)	51% (14, 88)	22
		Richard Toll	12% (1, 37)	32% (5, 74)	26
Specificity	Cattle	Barkedji	55% (5, 98)	51% (9, 92)	12
		Richard Toll	70% (16, 98)	69% (35, 93)	27
	Small ruminants	Barkedji	91% (73, 99)	81% (60, 94)	22
		Richard Toll	88% (65, 99)	84% (66, 95)	26

n = number of animals diagnosed

**Fig 1:**
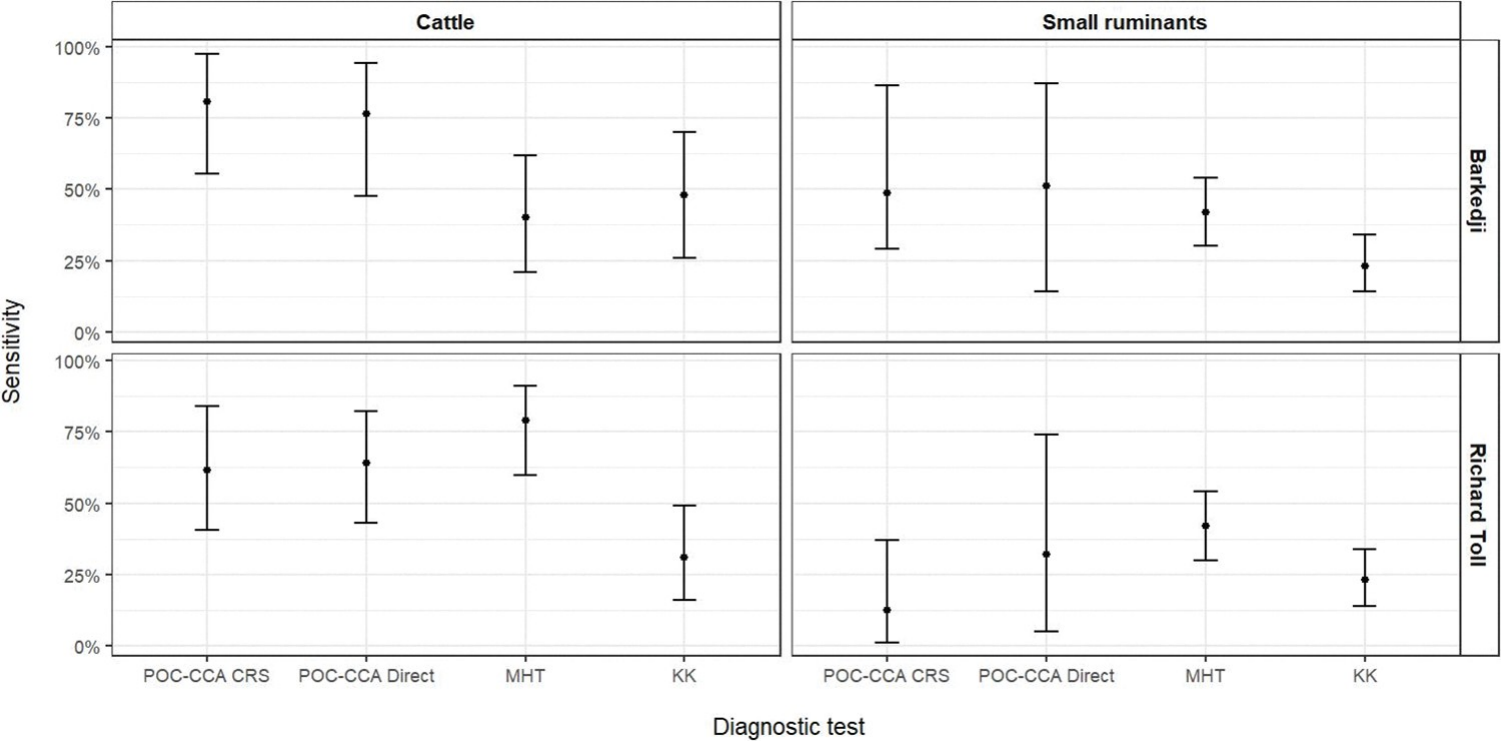
POC-CCA sensitivity (% median and 95% CrI). POC-CCA sensitivity based on composite reference standard (CRS), POC-CCA based on direct diagnostic (Direct), miracidial hatching technique (MHT) [8] and Kato Katz (KK) [8], by ruminant group and location.

Visual comparisons (Fig 1) of the accuracy results obtained in this study with those of the routinely-employed diagnostic techniques, MHT and KK, reported from Senegal [8], suggest that POC-CCA sensitivity was better than that of MHT and KK in Barkedji cattle and small ruminants, where *S*. *curassoni* was the main parasite species, and better than KK but worse than MHT in Richard Toll cattle, where *S*. *bovis* was the main parasite. POC-CCA sensitivity performed worse than MHT and KK in Richard Toll small ruminants.

#### Prior sensitivity analysis and comparison of accuracy in abattoir and live populations

The results of the prior sensitivity analyses indicate that the baseline prior models adopted were robust and that the priors were not exerting an unduly effect on the POC-CCA accuracy estimates (see Section 6, Table G in S1 Appendix).

Median POC-CCA accuracy in abattoir and live populations were similar (refer to Section 7, Table H in S1 Appendix), although their 95% CrI were wider than those of pooled samples.

## Discussion

In multi-host, multi-parasite systems, all reservoir hosts must be considered in order to achieve disease elimination. Accurate detection of *Schistosoma* spp. infection in animals would provide not only critical information to guide surveillance and inform control [2–4], but also help to improve livestock-keeping communities wellbeing, finances, and animal welfare [5, 7]. As WHO therefore calls for consideration of the need to treat livestock within Africa in order to minimize zoonotic transmission to humans, as well as for improved diagnostics in general [2–4], accurate diagnosis of schistosomiasis at both the individual and population levels is required for sustainable control programmes as well as assessing, and mitigating against, changes in drug efficacy. This study thereby evaluated the clinical performance of the commercially-available POC-CCA, a diagnostic test routinely used for the detection of the human intestinal parasite, *S*. *mansoni*, for intestinal *S*. *bovis*, *S*. *curassoni* and hybridized schistosomiasis infections within ruminant livestock of Senegal, West Africa.

Visual assessments of POC-CCA, MHT and KK sensitivity (see Fig 1) suggested that POC-CCA performance might have been associated both with host group and with parasite species. POC-CCA specificity in small ruminants was high (91%; 95% CrI: 77%, 99%) and did not seem to depend on parasite species. We are not aware of other studies in the literature reporting accuracy results for the detection of CCA in *S*. *bovis* or *S*. *curassoni* infections in livestock. However, CCA has been reported as being strongly associated with *S*. *bovis* worm and faecal egg counts in goats [54, 55] and CAA were detected in *S*. *mattheei* infected cows [56].

The test manufacturers have found POC-CCA accuracy to vary by parasite species, indicating that the test was particularly useful for the detection of human intestinal schistosomiasis caused by *S*. *mansoni*, and less so in the diagnosis of *S*. *haematobium* [57]. Kittur *et al*. suggested that *S*. *haematobium* and *S*. *mansoni* worms may produce different amounts of CCA or that *S*. *haematobium* may metabolise it more efficiently [58]. It is plausible that *S*. *bovis* and *S*. *curassoni* similarly have different patterns of CCA excretion and metabolic pathways, and thus respond differently in POC-CCA accuracy. If the maximum number of adult worms a host can carry falls beneath the test’s minimum level of detection, infected hosts would be misdiagnosed as uninfected. These maxima may differ with parasite species, resulting in differential misclassification. To our knowledge, the respective maximum number of worms each host can carry, by parasite species and ruminant group, has not been determined. The disparity between each host species’ test performance might be attributable to the factors such as consistency of urine, rate of metabolising CCA, or cross-reaction with other infections.

Our study also found that there were no differences in POC-CCA specificity in small ruminants across sites and parasite species, and that POC-CCA specificity was not affected by haematuria. This contrasts with results in humans, where POC-CCA specificity has been found to be affected by the host’s age (in pre-school aged children < 5 years), their pregnancy status, and whether or not they have haematuria or a urogenital infection [39, 59].

POC-CCA accuracy has been found to vary depending on production batch, raising questions regarding production quality control and calls for the optimisation and standardisation of production [60–62]. This is of particular relevance if POC-CCA is to become reliable a tool with which to determine whether to treat human and livestock populations [2–4].

### Limitations

Due to the absence of a gold-standard diagnostic test for livestock schistosomiasis, this study derived a composite reference standard (CRS) that was based on KK, MHT and UCAA results. The sensitivities of KK and MHT are relatively low, partly due to the technical difficulties associated with the management of large volumes of faecal material. These methods are highly specific, although misclassification of eggs and miracidia can occur. On the other hand, UCAA assays had been labelled as ultrasensitive for human schistosomiasis detection [50, 63], but have not been optimised for livestock. By combining these tests into a CRS we aimed to obtain a composite measure that was highly specific and moderately sensitive. However, we found eight cases where UCAA was negative and KK or MHT were positive, indicating that the high sensitivity UCAA test may not detect all the infections despite its lower limit of detection of 0.6 pg/mL CAA [63]. Given the variable quality of the urine samples, it was unfortunate that paired serum samples were not available to validate urine testing. CRS in diagnostic accuracy assessment can lead to biased estimates when CRSs are assumed to be error-free [64]. We addressed this potential source of bias by introducing uncertainty in the CRS classification through the adoption of CRS sensitivity and CRS specificity prior distributions, and by carrying out prior sensitivity analyses, that led to the conclusion that the estimates were stable. Our POC-CCA (CRS) results were similar to those obtained by direct diagnostic of abattoir ruminants, suggesting that they were robust with respect to CRS-associated bias. There is no consensus as to whether POC-CCA trace readings should be considered as representing a positive result or as a lack of infection. By considering trace as negative result, one would reduce the test sensitivity and increase its specificity, as fewer positive cases would be detected but more true negative cases would be identified. In this study we followed the recommendations in the literature for human schistosomiasis [65], and we considered trace results as positive infections. However, this may have resulted in an overestimation of sensitivity and underestimation of specificity of POC-CCA. The relatively low sample sizes for goats and sheep limited our ability to analyse goat and sheep data separately. The half-width of the credible intervals reported in this study was on average 20% from the median, except for specificity in cattle, where the width was greater. Lager sample sizes and samples from populations with a wider range of prevalence would have enabled us to calculate more precise estimates both for sensitivity and specificity. It is likely that POC-CCA accuracy in Barkedji and Richard Toll can be assimilated to accuracy for *S*. *curassoni* and *S*. *bovis* detection, respectively. However, this need to be further investigated. The observed high probabilities of sensitivity differences point to novel evidence of variation between host and parasite species that can inform the design and analyses of future diagnostic evaluations.

## Conclusions

Overall, our results indicate that the current POC-CCA developed for human use does represent a potential diagnostic tool for schistosomiasis in livestock populations. However, the observed variation in test performance across host species, parasite species and sites does have implications for the broader applicability of this diagnostic method, as it may hinder our ability to establish universally valid thresholds for disease prevalence that inform control programmes. Hence, the factors that determine test performance need to be investigated further so that region-specific guidelines could be derived if needed. Likewise, manufacturers quality control must be a foremost priority if POC-CCA diagnostic tests are implemented for the assessment of schistosomiasis, both in humans and animals. In order to move towards the interruption of transmission, the elimination of this zoonotic transmitted disease [2, 3] and to safeguard the welfare of livestock and the livelihoods of the communities that depend on them [5], ideally future work would focus on developing additional or optimised inexpensive livestock-specific POC-CCA tests that would enable us to formulate accurate assessments of disease prevalence.

## Supporting information

S1 Appendix For Sensitivity and specificity of human point-of-care circulating cathodic antigen (POC-CCA) test in African livestock for rapid diagnosis of schistosomiasis: A Bayesian latent class analysis. 1. Description of diagnostic tests. Table A. Summary of diagnostic tests and definitions of positive results. 2. Molecular analyses for Schistosoma species determination. 3. Statistical analysis. Table B. Diagnostic tests and covariance setting included in the models studied. Table C. Baseline priors used in multivariate modelling. 4. Observed prevalence. Table D. Summary statistics by diagnostic method: number of infected animals/number of animals examined (empirical prevalence), by site (S), ruminant group (R) and animal population (P). 5. Distribution of Schistosoma species. Table E. Number of animals genotyped and number of animals in each combination of Schistosoma species. 6. BLCM sensitivity. Table F. Priors adopted in the sensitivity analysis, modifying CRS priors (A) and prevalence priors (B). Table G. BLCM results from 2-tests independence analyses showing parameter median (95% CrI) and models’ deviance information criterium (DIC), for POC-CCA sensitivity (Se, %) and specificity (Sp, %), comparing baseline priors, CRS modified priors and prevalence modified priors. 7. Comparison of sensitivity and specificity in live and abattoir populations. 8. Models JAGS code. 9. References. 10. Data.
(DOCX)
