# Supplementary material for: Sensitivity and specificity of human point-of-care circulating cathodic antigen (POC-CCA) test in African livestock for rapid diagnosis of schistosomiasis: A Bayesian latent class analysis
Source: PLoS Negl Trop Dis. 2023 May 22;17(5):e0010739. doi: 10.1371/journal.pntd.0010739 (PMC10237635; doi:10.1371/journal.pntd.0010739)
Supplement: S1 Appendix — 1. Description of diagnostic tests. Table A. Summary of diagnostic tests and definitions of positive results. 2. Molecular analyses for Schistosoma species determination. 3. Statistical analysis. Table B. Diagnostic tests and covariance setting included in the models studied. Table C. Baseline priors used in multivariate modelling. 4. Observed prevalence. Table D. Summary statistics by diagnostic method: number of infected animals/number of animals examined (empirical prevalence), by site (S), ruminant group (R) and animal population (P). 5. Distribution of Schistosoma species. Table E. Number of animals genotyped and number of animals in each combination of Schistosoma species. 6. BLCM sensitivity. Table F. Priors adopted in the sensitivity analysis, modifying CRS priors (A) and prevalence priors (B). Table G. BLCM results from 2-tests independence analyses showing parameter median (95% CrI) and models’ deviance information criterium (DIC), for POC-CCA sensitivity (Se, %) and specificity (Sp, %), comparing baseline priors, CRS modified priors and prevalence modified priors. 7. Comparison of sensitivity and specificity in live and abattoir populations. 8. Models JAGS code. 9. References. 10. Data. (DOCX) [file pntd.0010739.s001.docx]

# S1 Appendix for *Sensitivity and specificity of human point-of-care circulating cathodic antigen (POC-CCA) test in African livestock for rapid diagnosis of schistosomiasis: a Bayesian latent class analysis.*

# Description of diagnostic tests

## Kato Katz technique (KK)

A small faecal sample (41.7 mg) is placed on a microscope slide, levelled, and covered with a piece of cellophane soaked overnight in glycerol solution. The slide is inverted and pressed firmly against the cellophane strip to spread faeces. The slide is left to dry for about an hour and examined under a microscope for the presence of *Schistosoma* eggs [1].

## Miracidial hatching technique (MHT)

Faecal samples are weighed (15g for cattle; 5g for small ruminants), passed through a 400μm metal sieve into a plastic container (*Schistosoma* eggs pass through) whilst rinsing with de-chlorinated water. The filtrate is then passed through both parts of a Pitchford funnel, and any eggs present sink to the bottom of the Pitchford funnel. Eggs are dispensed into a specimen pot with additional de-chlorinated water and placed under light to facilitate hatching of eggs into miracidia. Samples are then examined microscopically for the presence of miracidia. Tissue samples are macerated before being processed in the same way as faeces. Technique adapted from Yu *et al.* 2007 [2].

## Point-of-care circulating cathodic antigen test POC-CCA

The instruction supplied by the manufactures for the early generation of tests were employed [3]. In brief, one drop of urine and one drop of buffer were placed into the circular well of the test cassette and the results were read exactly 20 minutes later.

## Up-converting reporter particle based lateral flow (UCP-LF) assay formats to measure circulating cathodic/anodic antigen in urine (UCCA/UCAA)

Urine was stored and shipped to Leiden University Medical Centre at -20 °C. All urine samples were tested for both antigens using respectively 10 and 417μL urine for CCA and CAA (UCCA10 and UCAA*hT*417 assay formats [4]). All samples were extracted with trichloroacetic acid (TCA) at an end volume of 2% (v/v) and centrifuged to remove precipitate leaving the carbohydrate component in the TCA-supernatant [5]. The supernatant was concentrated down to 20 μL (UCAA*hT*417 only) and subsequently the equivalent of 10 and 417 μL TCA-extracted urine mixed with 100 μL assay buffer (HSLF: 200 mM Tris pH 8, 270 mM NaCl, 1% BSA (w/v), 0.5% (v/v) Tween-20 containing 100 ng up-converting reporter particles (UCP) coated with the appropriate antibody (anti-CCA or anti-CAA [5]) and incubated for 1h in a thermo-shaker (37°C). The mixture was applied to the appropriate LF strip and immunochromatography allowed to proceed until strips were dry. Strips were scanned with an adapted Packard FluoroCount microtitre plate reader utilizing an infrared laser (980 nm) for excitation of the UCP. Emission signals (540 nm) were measured as relative fluorescent units (RFUs) at both the Test (T) and Flow Control (FC) lines [6]. Sample concentrations were determined by fitting the T/FC ratio obtained to a standard reference series, an adult worm TCA extract containing 3% w/w of CAA or CCA spiked in negative urine [5].

Cut-offs for the UCCA10 and UCAA*hT*417 assays were set to 2400 and 0.6 pg/mL, respectively, as previously determined with spiked human urine. CCA levels in urine are generally at least 100 to 1000 fold higher than CAA, and due to a biological background in urine for CCA (Lewis X structure) it was agreed that testing at a higher volume of urine would not be useful.

## Hemastix

Hemastix reagent strips are employed to test for presence of blood in urine (Siemens Healthcare Diagnostics, Surrey, UK). The strip is dipped into the urine and the change in colour is visually assessed. No change in colour indicates that there is no blood in urine or that the amount is below the level of detection. Colour changes can be compared with reference values. The three positive values (+, ++, +++) recorded for livestock animals were combined into a single positive category.

## Table A. Summary of diagnostic tests and definitions of positive results

| **Short code** | **Diagnostic** | **Which populations** | **Sample used** | **Metric** | **Definition of positive** |
| --- | --- | --- | --- | --- | --- |
| KK | Kato-Katz thick smear technique | Abattoir and live animals | 2 x slides from a single faecal sample | Number of *Schistosoma* eggs detectable by microscopy | One or more eggs found on the slides |
| MHT | Miracidial hatching technique (faecal) | Abattoir and live animals | Faeces | Number of swimming miracidia | One or more swimming miracidia |
| POC-CCA | Point-of-care circulating cathodic antigen test (trace considered positive) | Abattoir and live animals | Urine | Intensity of band against a reference | Trace/+1/+2/+3 |
| UCCA10 | Up-converting phosphor-lateral flow circulating cathodic antigen assay performed with 10 μL of urine | Abattoir and live animals | Urine | Laboratory test reader (strip scanner) | >2400 pg/mL CCA |
| UCAA  *hT*417 | Up-converting phosphor-lateral flow circulating anodic antigen assay performed with 10 μL of urine | Abattoir and live animals | Urine | Laboratory test reader (strip scanner) | >0.6 pg/mL CAA |
| Hemastix | Hemastix reagent strip | Abattoir and live animals | Urine | Intensity of band against a reference provided by the manufacturer (presumptive test) | +1/+2/+3 |
| Adult worms | Adult worms in blood vessels | Abattoir | Carcass: mesenteric blood vessels | Number of single and paired worms | One or more worms found |
| Organs MHT | Miracidial hatching technique (organs and faeces) | Abattoir | Carcass: lung and liver  Faeces | Number of swimming miracidia | One or more swimming miracidia |

# Molecular analyses for *Schistosoma* species determination

Molecular analyses performed on *Schistosoma* parasitological samples are detailed in [7]. Briefly, DNA was extracted from adult worms stored in RNA-later following DNA extraction kit manufacturer guidelines and eluted from miracidia from the Whatman FTA classic cards [8]. *Schistosoma* DNA extracts were characterized by amplification of a partial fragment of the mitochondrial cytochrome oxidase subunit 1 (*cox1*) and the complete nuclear ribosomal DNA internal transcribed spacer (ITS). PCR fragments were sequenced by Eurofins GATC Genomics (Cologne, Germany) using original PCR primers. DNA sequences were manually edited and assembled using CodonCode Aligner (v.7.0.1) (Centerville, USA) and compared with *Schistosoma* reference sequences for species identification [7].

# Statistical analysis

## Latent Class Models

Latent Class Models (LCMs) comprise a range of probabilistic tools that are often used to assess diagnostic test accuracy when the true status of a disease cannot be ascertain with certainty, e.g. when there is no “gold standard” [9]. LCM consider the true status of the disease as a “latent” categorical variable and through statistical modelling, relates this latent variable to the outcomes of several imperfect diagnostic tests, thus enabling us to estimate the test accuracy [10]. Bayesian Latent Class Models (BLCM) make inferences within a Bayesian framework, and in doing so they overcome some of the limitations associated with the frequentist paradigm. In particular, BLCMs avoid the need to constrain parameter ranges to make model identifiable [11]. Conversely, BLCM assume prior distributions for the parameters being examined, that when combined with the data, lead to posterior distribution from which parameter summary statistics can be derived [11]. Table B shows the models assessed in this study, the diagnostic tests that were included and whether the covariance was assumed to be zero or not.

## Table B. Diagnostic tests and covariance setting included in the models studied.

| **Model** | **Two-Test Independence** | **Two-Test Dependence** | **Three-Test** |
| --- | --- | --- | --- |
| **Diagnostic test** | POC-CCA | POC-CCA | POC-CCA |
|  | CRS* | CRS* | CRS* |
|  |  |  | UCCA |
| **Covariance (Cov)** | Cov(POC-CCA, CRS) = 0 | Cov(POC-CCA, CRS) ≠ 0 | Cov(POC-CCA, CRS) = 0 |
|  |  |  | Cov(POC-CCA, UCCA) ≠ 0 |

*CRS(+) = UCAA(+) or MHT(+) or KK(+); CRS(-) = UCAA(-) and MHT(-) and KK(-)

## Software setting

BLCM analyses were implemented by Markov Chain Monte Carlo (MCMC) simulations and were carried out in R v. 4.0.5 with JAGS by means of the “rjags” package v. 4-10 [12-14]. Models convergence and autocorrelation were assessed by means of the package “CODA” v. 0.19-4 and “mcmcplots” [15, 16]. Three chains were generated, and their convergence was assessed by the Gelman-Rubin diagnostic and by visual inspection of trace plots [17]. In order to determine the number of iterations needed, Raftery and Lewis diagnostic tests were performed with default settings [18, 19]. A total of 50000 iterations were used with a thinning interval of 10 and 5000 burn-in iterations resulting in (50000 – 5000)/10*3 = 13,500 iterations. Autocorrelation was assessed visually after executing the “CODA” package command “autocorr.plot”. The outputs of interest were the median and the 95% credible intervals for each parameter.

## Table C. Baseline priors used in multivariate modelling.

| **Test/Group** | **Parameter** | **Mode** | **95% Certainty that true value of mode is greater(*smaller) than:** | **Baseline priors** |
| --- | --- | --- | --- | --- |
| POC-CCA | Sensitivity | 0.50 | 0.05 | Beta(1, 1) |
|  | Specificity | 0.50 | 0.05 | Beta(1, 1) |
| UCCA | Sensitivity | 0.60 | 0.30 | Beta(4.84, 3.56) |
|  | Specificity | 0.75 | 0.50 | Beta(9.63,3.88) |
| CRS | Sensitivity | 0.75 | 0.60 | Beta(23.57, 8.52) |
|  | Specificity | 0.95 | 0.70 | Beta(10.78, 1.51) |
| Cattle | Prevalence | 0.60 | 0.40 | Beta(10.90, 7.60) |
| Small ruminants | Prevalence | 0.35 | 0.55* | Beta(7.03, 12.20) |

POC-CCA= point-of-care cathodic circulating antigen; CRS: composite reference standard; UCCA: Up-converting phosphor-lateral flow circulating cathodic antigen assay.

# Observed prevalence

## Table D. Summary statistics by diagnostic method: number of infected animals/number of animals examined (empirical prevalence), by site (S), ruminant group (R) and animal population (P). C = cattle, SR = small ruminants; B = Barkedji, R = Richard Toll; A = abattoir, L = live animals.

| **S** | **R** | **P** | **POC-CCA** | **CRS** | **UCAA** | **UCCA** | **MHT** | **Kato Katz** |
| --- | --- | --- | --- | --- | --- | --- | --- | --- |
| B | C | A | 9/12  (75%) | 11/13  (85%) | 10/13  (77%) | 5/13  (38%) | 5/12  (42%) | 6/12  (50%) |
|  |  | L | 5/6  (83%) | 8/8  (100%) | 8/8  (100%) | 6/8  (75%) | 0/4  (0%) | 0/8  (0%) |
|  | SR | A | 5/22  (23%) | 9/22  (41%) | 9/22  (41%) | 3/22  (14%) | 2/22  (9%) | 0/21  (0%) |
|  |  | L | 20/67  (30%) | 40/68  (59%) | 36/68  (53%) | 35/68  (51%) | 5/46  (11%) | 6/66  (9%) |
| R | C | A | 15/27  (56%) | 23/28  (82%) | 22/28  (79%) | 16/28  (57%) | 13/27  (48%) | 10/27  (37%) |
|  |  | L | 4/7  (57%) | 6/7  (86%) | 5/7  (71%) | 2/7  (29%) | 6/7  (86%) | 2/7  (29%) |
|  | SR | A | 4/26  (15%) | 17/26  (65%) | 16/26  (62%) | 5/26  (19%) | 1/25  (4%) | 2/25  (8%) |
|  |  | L | 1/23  (4%) | 12/23  (52%) | 12/23  (52%) | 5/23  (22%) | 2/22  (9%) | 0/22  (0%) |

POC-CCA= point-of-care cathodic circulating antigen; CRS: composite reference standard; UCAA: Up-converting phosphor-lateral flow circulating anodic antigen assay; UCCA: Up-converting phosphor-lateral flow circulating cathodic antigen assay; MHT: miracidial hatching technique; KK: Kato Katz technique.

# Distribution of *Schistosoma* species

The distributions of the numbers of animals with genotyped miracidia are presented in Table E. Owing to the limited number of miracidia that were genotyped per animal, it was not possible to derive definitive conclusions on co-occurrence of parasites at the animal level. The results presented here suggest that both species and hybrids coexisted in both locations, and that *S. curassoni* predominated in Barkedji whilst *S. bovis* dominated in Richard Toll. In this sub-population, hybrids were found in cattle but not in small ruminants.

## Table E. Number of animals genotyped and number of animals in each combination of *Schistosoma* species. Sb only = only *S. bovis* miracidia genotyped in the animals examined. Sc stands for *S. curassoni*. Co-occurrence of different combinations of species in an animal is denoted as: “Sb & Sc only”, “Sb & hybrids only”, “Sc & hybrids only” and “Sb & Sc & hybrids”. B Barkedji, R=Richard Toll.

| **Site** | **Source** | **Ruminant**  **Group** | **Sample**  **Total** | **Genotyped** | **Sb only** | **Sc only** | **Hybrids only** | **Sb & Sc only** | **Sb & hybrids only** | **Sc & hybrids only** | **Sb & Sc & hybrids** |
| --- | --- | --- | --- | --- | --- | --- | --- | --- | --- | --- | --- |
| B | Abattoir | Bovine | 13 | 10 |  | 5 |  |  |  | 3 | 2 |
|  |  | Goat | 19 | 2 |  | 2 |  |  |  |  |  |
|  |  | Sheep | 3 | 1 |  | 1 |  |  |  |  |  |
|  |  | Small ruminants | 22 | 3 |  | 3 |  |  |  |  |  |
|  | Live | Bovine | 8 | 0 |  |  |  |  |  |  |  |
|  |  | Goat | 1 | 1 |  | 1 |  |  |  |  |  |
|  |  | Sheep | 57 | 3 |  | 3 |  |  |  |  |  |
|  |  | Small ruminants | 58 | 4 |  | 4 |  |  |  |  |  |
| R | Abattoir | Bovine | 28 | 18 | 14 | 1 |  | 1 |  | 1 | 1 |
|  |  | Goat | 25 | 2 | 2 |  |  |  |  |  |  |
|  |  | Sheep | 1 | 0 |  |  |  |  |  |  |  |
|  |  | Small ruminants | 26 | 2 | 2 |  |  |  |  |  |  |
|  | Live | Bovine | 7 | 6 | 6 |  |  |  |  |  |  |
|  |  | Goat | 2 | 1 | 1 |  |  |  |  |  |  |
|  |  | Sheep | 21 | 1 | 1 |  |  |  |  |  |  |
|  |  | Small ruminants | 23 | 2 | 2 |  |  |  |  |  |  |

# BLCM sensitivity

## Table F. Priors adopted in the sensitivity analysis, modifying CRS priors (A) and prevalence priors (B).

| **Analysis** | **Parameter** | **Mode** | **95% Certainty that true value of mode is greater(*smaller) than:** | **Priors** |
| --- | --- | --- | --- | --- |
| A. CRS | Sensitivity | 0.55 | 0.4 | Beta(16.94, 14.04) |
|  | Specificity | 0.75 | 0.5 | Beta(9.62, 3.88) |
| B. Prevalence | Cattle | 0.60 | 0.30 | Beta(4.84, 3.56) |
|  | Small ruminants | 0.35 | 0.65* | Beta(3.48, 5.61) |

CRS = composite reference standard

Table G shows the results for the 2-tests independence model fitted under baseline priors, CRS modified priors and prevalence modified priors. In cattle at both sites, the lowest DICs corresponded to the model in which the prevalence priors had been modified. The point estimates and 95% CrI in all cases were very similar, suggesting that the impact of the change in priors on the accuracy results was minor.

In the case of small ruminants there were differences between Barkedji and Richard Toll. In the former, the baseline model and the prevalence modified models fitted the data best and their accuracy estimates were very similar. Although in Richard Toll the best fitting model was the one with modified CRS priors, the accuracy estimates obtained with the three prior configurations were very similar. Based on these sensitivity tests, there was no reason to suspect that the priors are exerting an unduly effect on the accuracy estimates.

## Table G. BLCM results from 2-tests independence analyses showing parameter median (95% CrI) and models’ deviance information criterium (DIC), for POC-CCA sensitivity (Se, %) and specificity (Sp, %), comparing baseline priors, CRS modified priors and prevalence modified priors.

| **Ruminant** | **Site** | **Variable** | **Baseline Priors** | **CRS accuracy** | **Prevalence** |
| --- | --- | --- | --- | --- | --- |
| Cattle | B | Se | 81 (55, 98) | 81 (44, 99) | 80 (56, 96) |
|  |  | Sp | 55 (5, 98)* | 44 (2, 96)* | 55 (4, 98)* |
|  |  | DIC | 47.96 | 54.32 | 43.75 |
|  | R | Se | 62 (41, 84) | 62 (21, 94) | 60 (40, 80) |
|  |  | Sp | 70 (16, 98)* | 61 (5, 98)* | 69 (11, 98)* |
|  |  | DIC | 54.27 | 61.08 | 50.18 |
| Small Ruminants | B | Se | 49 (29, 87) | 56 (11, 95) | 45 (28, 82) |
|  |  | Sp | 91 (73, 99) | 91 (60, 100) | 91 (74, 100) |
|  |  | DIC | 55.92 | 61.56 | 55.33 |
|  | R | Se | 12 (1, 37) | 15 (1, 58) | 12 (1, 34) |
|  |  | Sp | 88 (65, 99) | 90 (67, 99) | 87 (59, 99) |
|  |  | DIC | 46.53 | 43.54 | 45.22 |

B = Barkedji, R = Richard Toll

* Sample sizes were too low to derive precise estimates.

# Comparison of sensitivity and specificity in live and abattoir populations

## Table H. POC-CCA sensitivity (%) and specificity (%) in live and abattoir animal populations combined (Live & abattoir), live animals only (Live) and abattoir animals only (Abattoir), by ruminant group and site.

| **Parameter** | **Ruminant** | **Site** | **Live & abattoir** | **Live** | **Abattoir** |
| --- | --- | --- | --- | --- | --- |
| Sensitivity (%) | Cattle | Barkedji | 81 (55, 98) | 78 (40, 98) | 78 (46, 97) |
|  |  | Richard Toll | 62 (40, 84) | 63 (24, 95) | 60 (36, 83) |
|  | Small ruminants | Barkedji | 49 (29, 87) | 47 (25, 87) | 50 (16, 91) |
|  |  | Richard Toll | 12 (1, 37) | 12 (1, 46) | 14 (1, 47) |
| Specificity (%) | Cattle | Barkedji | 55 (5, 98) | 48 (2, 97) | 54 (5, 97) |
|  |  | Richard Toll | 70 (16, 98) | 66 (7, 98) | 62 (9, 98) |
|  | Small ruminants | Barkedji | 91 (73, 99) | 87 (63, 99) | 90 (65, 100) |
|  |  | Richard Toll | 88 (65, 99) | 94 (69, 100) | 75 (36, 97) |

# Models JAGS code

## JAGS code for two-test independence/dependence models [20].

model{

### Population 1

# Likelihood

y1[1:4] ~ dmulti(p1[1:4], n1)

p1[1] <- pi1 * (SePOC1 * SeCRS1 + cov_Se1) + (1-pi1) * ((1-SpPOC1) * (1-SpCRS1) + cov_Sp1) # ++

p1[2] <- pi1 * (SePOC1 * (1-SeCRS1) - cov_Se1) + (1-pi1) * ((1-SpPOC1) * SpCRS1 - cov_Sp1) # +-

p1[3] <- pi1 * ((1-SePOC1) * SeCRS1 - cov_Se1) + (1-pi1) * (SpPOC1 * (1-SpCRS1) - cov_Sp1) # -+

p1[4] <- pi1 * ((1-SePOC1) * (1-SeCRS1) + cov_Se1) + (1-pi1) * (SpPOC1 * SpCRS1 + cov_Sp1) # --

# Priors

pi1 ~ dbeta(p1a, p1b)

SePOC1 ~ dbeta(SePOCa, SePOCb)

SpPOC1 ~ dbeta(SpPOCa, SpPOCb)

SeCRS1 ~ dbeta(SeCRSa, SeCRSb)

SpCRS1 ~ dbeta(SpCRSa, SpCRSb)

#cov_Se1_a <- max(-(1 - SePOC1)*(1 - SeCRS1), - SePOC1 * SeCRS1) # uncomment for dependence model

#cov_Se1_b <- min(SePOC1 * (1 - SeCRS1), (1 - SePOC1) * SeCRS1) # uncomment for dependence model

cov_Se1 <- 0 # ~ dunif(cov_Se1_a, cov_Se1_b) # comment for dependence model

#cov_Sp1_a <- max(-(1 - SpPOC1)*(1 - SpCRS1), -SpPOC1 * SpCRS1) # uncomment for dependence model

#cov_Sp1_b <- min(SpPOC1 * (1 - SpCRS1), (1 - SpPOC1) * SpCRS1) # uncomment for dependence model

cov_Sp1 <- 0 # ~ dunif(cov_Sp1_a, cov_Sp1_b) # comment for dependence model

#####

### Population 2

# Likelihood

y2[1:4] ~ dmulti(p2[1:4], n2)

p2[1] <- pi2 * (SePOC2 * SeCRS2 + cov_Se2) + (1-pi2) * ((1-SpPOC2) * (1-SpCRS2) + cov_Sp2) # ++

p2[2] <- pi2 * (SePOC2 * (1-SeCRS2) - cov_Se2) + (1-pi2) * ((1-SpPOC2) * SpCRS2 - cov_Sp2) # +-

p2[3] <- pi2 * ((1-SePOC2) * SeCRS2 - cov_Se2) + (1-pi2) * (SpPOC2 * (1-SpCRS2) - cov_Sp2) # -+

p2[4] <- pi2 * ((1-SePOC2) * (1-SeCRS2) + cov_Se2) + (1-pi2) * (SpPOC2 * SpCRS2 + cov_Sp2) # --

# Priors

pi2 ~ dbeta(p2a, p2b)

SePOC2 ~ dbeta(SePOCa, SePOCb)

SpPOC2 ~ dbeta(SpPOCa, SpPOCb)

SeCRS2 ~ dbeta(SeCRSa, SeCRSb)

SpCRS2 ~ dbeta(SpCRSa, SpCRSb)

#cov_Se2_a <- max(-(1 - SePOC2)*(1 - SeCRS2), -SePOC2 * SeCRS2) # uncomment for dependence model

#cov_Se2_b <- min(SePOC2 * (1 - SeCRS2), (1 - SePOC2) * SeCRS2) # uncomment for dependence model

cov_Se2 <- 0 # ~ dunif(cov_Se2_a, cov_Se2_b) # comment for dependence model

#cov_Sp2_a <- max(-(1 - SpPOC2)*(1 - SpCRS2), -SpPOC2 * SpCRS2) # uncomment for dependence model

#cov_Sp2_b <- min(SpPOC2 * (1 - SpCRS2), (1 - SpPOC2) * SpCRS2) # # uncomment for dependence model

cov_Sp2 <- 0 # ~ dunif(cov_Sp2_a, cov_Sp2_b) # comment for dependence model

### Comparison between populations 1 and 2

zSe <- step(SePOC1 - SePOC2) # Differences in sensitivity of POC-CCA

zSp <- step(SpPOC1 - SpPOC2)

####

### Both populations together

# Likelihood

y3[1:4] ~ dmulti(p3[1:4], n3)

p3[1] <- pi3 * (SePOC * SeCRS + cov_Se) + (1-pi3) * ((1-SpPOC) * (1-SpCRS) + cov_Sp) # ++

p3[2] <- pi3 * (SePOC * (1-SeCRS) - cov_Se) + (1-pi3) * ((1-SpPOC) * SpCRS - cov_Sp) # +-

p3[3] <- pi3 * ((1-SePOC) * SeCRS - cov_Se) + (1-pi3) * (SpPOC * (1-SpCRS) - cov_Sp) # -+

p3[4] <- pi3 * ((1-SePOC) * (1-SeCRS) + cov_Se) + (1-pi3) * (SpPOC * SpCRS + cov_Sp) # --

## Priors

pi3 ~ dbeta(p3a, p3b)

SePOC ~ dbeta(SePOCa, SePOCb)

SpPOC ~ dbeta(SpPOCa, SpPOCb)

SeCRS ~ dbeta(SeCRSa, SeCRSb)

SpCRS ~ dbeta(SpCRSa, SpCRSb)

#cov_Se_a <- max(-(1 - SePOC)*(1 - SeCRS), - SePOC * SeCRS) # uncomment for dependence model

#cov_Se_b <- min(SePOC * (1 - SeCRS), (1 - SePOC) * SeCRS) # uncomment for dependence model

cov_Se <- 0 # ~ dunif(cov_Se_a, cov_Se_b) # comment for dependence model

#cov_Sp_a <- max(-(1 - SpPOC)*(1 - SpCRS), - SpPOC * SpCRS) # uncomment for dependence model

#cov_Sp_b <- min(SpPOC * (1 - SpCRS), (1 - SpPOC) * SpCRS) # uncomment for dependence model

cov_Sp <- 0 # ~ dunif(cov_Sp_a, cov_Sp_b) # comment for dependence model

}

## JAGS code for three-test model [21].

model {

### Population 1

# Likelihood

y1[1:8] ~ dmulti(p1[1:8], n1)

p1[1] <- pi1*(SePOC1*SeUCCA1+cov_Se1)*SeCRS1 + (1-pi1)*((1-SpPOC1)*(1-SpUCCA1)+cov_Sp1)*(1-SpCRS1)

p1[2] <- pi1*(SePOC1*SeUCCA1+cov_Se1)*(1-SeCRS1) + (1-pi1)*((1-SpPOC1)*(1-SpUCCA1)+cov_Sp1)*(SpCRS1)

p1[3] <- pi1*(SePOC1*(1-SeUCCA1)-cov_Se1)*SeCRS1 + (1-pi1)*((1-SpPOC1)*SpUCCA1-cov_Sp1)*(1-SpCRS1)

p1[4] <- pi1*(SePOC1*(1-SeUCCA1)-cov_Se1)*(1-SeCRS1) + (1-pi1)*((1-SpPOC1)*SpUCCA1-cov_Sp1)*(SpCRS1)

p1[5] <- pi1*((1-SePOC1)*SeUCCA1-cov_Se1)*SeCRS1 + (1-pi1)*(SpPOC1*(1-SpUCCA1)-cov_Sp1)*(1-SpCRS1)

p1[6] <- pi1*((1-SePOC1)*SeUCCA1-cov_Se1)*(1-SeCRS1) + (1-pi1)*(SpPOC1*(1-SpUCCA1)-cov_Sp1)*SpCRS1

p1[7] <- pi1*((1-SePOC1)*(1-SeUCCA1)+cov_Se1)*SeCRS1 + (1-pi1)*(SpPOC1*SpUCCA1+cov_Sp1)*(1-SpCRS1)

p1[8] <- pi1*((1-SePOC1)*(1-SeUCCA1)+cov_Se1)*(1-SeCRS1)+ (1-pi1)*(SpPOC1*SpUCCA1+cov_Sp1)*SpCRS1

# Priors

pi1 ~ dbeta(p1a, p1b)

SePOC1 ~ dbeta(SePOCa, SePOCb)

SpPOC1 ~ dbeta(SpPOCa, SpPOCb)

SeUCCA1 ~ dbeta(SeUCCAa,SeUCCAb)

SpUCCA1 ~ dbeta(SpUCCAa, SpUCCAb)

SeCRS1 ~ dbeta(SeCRSa, SeCRSb)

SpCRS1 ~ dbeta(SpCRSa, SpCRSb)

cov_Se1_a <- (SePOC1 - 1)*(1 - SeUCCA1)

cov_Se1_b <- min(SePOC1,SeUCCA1) - SePOC1*SeUCCA1

cov_Se1 ~ dunif(cov_Se1_a, cov_Se1_b)

cov_Sp1_a <- (SpPOC1 - 1)*(SpUCCA1)

cov_Sp1_b <- min(SpPOC1,SpUCCA1) - SpPOC1*SpUCCA1

cov_Sp1 ~ dunif(cov_Sp1_a, cov_Sp1_b)

#####

### Population 2

# Likelihood

y2[1:8] ~ dmulti(p2[1:8], n2)

p2[1] <- pi2*(SePOC2*SeUCCA2+cov_Se2)*SeCRS2 + (1-pi2)*((1-SpPOC2)*(1-SpUCCA2)+cov_Sp2)*(1-SpCRS2)

p2[2] <- pi2*(SePOC2*SeUCCA2+cov_Se2)*(1-SeCRS2) + (1-pi2)*((1-SpPOC2)*(1-SpUCCA2)+cov_Sp2)*(SpCRS2)

p2[3] <- pi2*(SePOC2*(1-SeUCCA2)-cov_Se2)*SeCRS2 + (1-pi2)*((1-SpPOC2)*SpUCCA2-cov_Sp2)*(1-SpCRS2)

p2[4] <- pi2*(SePOC2*(1-SeUCCA2)-cov_Se2)*(1-SeCRS2) + (1-pi2)*((1-SpPOC2)*SpUCCA2-cov_Sp2)*(SpCRS2)

p2[5] <- pi2*((1-SePOC2)*SeUCCA2-cov_Se2)*SeCRS2 + (1-pi2)*(SpPOC2*(1-SpUCCA2)-cov_Sp2)*(1-SpCRS2)

p2[6] <- pi2*((1-SePOC2)*SeUCCA2-cov_Se2)*(1-SeCRS2) + (1-pi2)*(SpPOC2*(1-SpUCCA2)-cov_Sp2)*SpCRS2

p2[7] <- pi2*((1-SePOC2)*(1-SeUCCA2)+cov_Se2)*SeCRS2 + (1-pi2)*(SpPOC2*SpUCCA2+cov_Sp2)*(1-SpCRS2)

p2[8] <- pi2*((1-SePOC2)*(1-SeUCCA2)+cov_Se2)*(1-SeCRS2)+ (1-pi2)*(SpPOC2*SpUCCA2+cov_Sp2)*SpCRS2

# Priors

pi2~dbeta(p2a, p2b)

SePOC2~dbeta(SePOCa, SePOCb)

SpPOC2~dbeta(SpPOCa, SpPOCb)

SeUCCA2~dbeta(SeUCCAa,SeUCCAb)

SpUCCA2~dbeta(SpUCCAa, SpUCCAb)

SeCRS2~dbeta(SeCRSa, SeCRSb)

SpCRS2~dbeta(SpCRSa, SpCRSb)

cov_Se2_a <- (SePOC2 - 1)*(1 - SeUCCA2)

cov_Se2_b <- min(SePOC2,SeUCCA2) - SePOC2*SeUCCA2

cov_Se2 ~ dunif(cov_Se2_a, cov_Se2_b)

cov_Sp2_a <- (SpPOC2 - 1)*(1 - SpUCCA2)

cov_Sp2_b <- min(SpPOC2,SpUCCA2) - SpPOC2*SpUCCA2

cov_Sp2 ~ dunif(cov_Sp2_a, cov_Sp2_b)

#### Comparison between populations 1 and 2

zSe <- step(SePOC1 - SePOC2)

zSp <- step(SpPOC1 - SpPOC2)

####

### Both populations together

# Likelihood

y3[1:8] ~ dmulti(p3[1:8], n3)

p3[1] <- pi3*(SePOC*SeUCCA+cov_Se)*SeCRS + (1-pi3)*((1-SpPOC)*(1-SpUCCA)+cov_Sp)*(1-SpCRS)

p3[2] <- pi3*(SePOC*SeUCCA+cov_Se)*(1-SeCRS) + (1-pi3)*((1-SpPOC)*(1-SpUCCA)+cov_Sp)*(SpCRS)

p3[3] <- pi3*(SePOC*(1-SeUCCA)-cov_Se)*SeCRS + (1-pi3)*((1-SpPOC)*SpUCCA-cov_Sp)*(1-SpCRS)

p3[4] <- pi3*(SePOC*(1-SeUCCA)-cov_Se)*(1-SeCRS) + (1-pi3)*((1-SpPOC)*SpUCCA-cov_Sp)*(SpCRS)

p3[5] <- pi3*((1-SePOC)*SeUCCA-cov_Se)*SeCRS + (1-pi3)*(SpPOC*(1-SpUCCA)-cov_Sp)*(1-SpCRS)

p3[6] <- pi3*((1-SePOC)*SeUCCA-cov_Se)*(1-SeCRS) + (1-pi3)*(SpPOC*(1-SpUCCA)-cov_Sp)*SpCRS

p3[7] <- pi3*((1-SePOC)*(1-SeUCCA)+cov_Se)*SeCRS + (1-pi3)*(SpPOC*SpUCCA+cov_Sp)*(1-SpCRS)

p3[8] <- pi3*((1-SePOC)*(1-SeUCCA)+cov_Se)*(1-SeCRS)+ (1-pi3)*(SpPOC*SpUCCA+cov_Sp)*SpCRS

# Priors

pi3~dbeta(p3a, p3b)

SePOC ~ dbeta(SePOCa, SePOCb)

SpPOC ~ dbeta(SpPOCa, SpPOCb)

SeUCCA ~ dbeta(SeUCCAa,SeUCCAb)

SpUCCA ~ dbeta(SpUCCAa, SpUCCAb)

SeCRS ~ dbeta(SeCRSa, SeCRSb)

SpCRS ~ dbeta(SpCRSa, SpCRSb)

# Covariance

cov_Se_a <- (SePOC - 1)*(1 - SeUCCA)

cov_Se_b <- min(SePOC, SeUCCA) - SePOC*SeUCCA

cov_Se ~ dunif(cov_Se_a, cov_Se_b)

cov_Sp_a <- (SpPOC - 1)*(1 - SpUCCA)

cov_Sp_b <- min(SpPOC, SpUCCA) - SpPOC*SpUCCA

cov_Sp ~ dunif(cov_Sp_a, cov_Sp_b)

}

# References

1. World Health Organization. Bench aids for the diagnosis of intestinal parasites. 2nd Edition ed2019.

2. Yu JM, de Vlas SJ, Jiang QW, Gryseels B. Comparison of the Kato-Katz technique, hatching test and indirect hemagglutination assay (IHA) for the diagnosis of *Schistosoma japonicum* infection in China. Parasitol Int. 2007;56(1):45-9. doi: 10.1016/j.parint.2006.11.002.

3. Rapid Medical Diagnostics. Schisto POC-CCA Rapid test for qualitative detection of Bilharzia (Schistosomiasis) 2018 [02/03/2022]. Available from: <https://www.rapid-diagnostics.com/>.

4. Corstjens PLAM, De Dood CJ, Knopp S, Clements MN, Ortu G, Umulisa I, et al. Circulating Anodic Antigen (CAA): A Highly Sensitive Diagnostic Biomarker to Detect Active *Schistosoma* Infections—Improvement and Use during SCORE. Am J Trop Med Hyg. 2020;103(1_Suppl):50-7. doi: 10.4269/ajtmh.19-0819.

5. Corstjens PLAM, De Dood CJ, Kornelis D, Tjon Kon Fat EM, Wilson RA, Kariuki TM, et al. Tools for diagnosis, monitoring and screening of *Schistosoma* infections utilizing lateral-flow based assays and upconverting phosphor labels. Parasitology. 2014;141(14):1841-55. doi: 10.1017/s0031182014000626.

6. Corstjens P, Zuiderwijk M, Brink A, Li S, Feindt H, Niedbala RS, et al. Use of up-converting phosphor reporters in lateral-flow assays to detect specific nucleic acid sequences: a rapid, sensitive DNA test to identify human papillomavirus type 16 infection. Clin Chem. 2001;47(10):1885-93. doi: 10.1093/clinchem/47.10.1885. PubMed PMID: 11568115.

7. Léger E, Borlase A, Fall CB, Diouf ND, Diop SD, Yasenev L, et al. Prevalence and distribution of schistosomiasis in human, livestock, and snail populations in northern Senegal: a One Health epidemiological study of a multi-host system. Lancet Planet Health. 2020;4(8):e330-e42. doi: 10.1016/S2542-5196(20)30129-7.

8. Webster BL, Rabone M, Pennance T, Emery AM, Allan F, Gouvras A, et al. Development of novel multiplex microsatellite polymerase chain reactions to enable high-throughput population genetic studies of *Schistosoma haematobium*. Parasit Vectors. 2015;8(1). doi: 10.1186/s13071-015-1044-6.

9. Pepe MS, Janes H. Insights into latent class analysis of diagnostic test performance. Biostatistics. 2007;8(2):474-84. doi: 10.1093/biostatistics/kxl038.

10. van Smeden M, Naaktgeboren CA, Reitsma JB, Moons KG, de Groot JA. Latent class models in diagnostic studies when there is no reference standard--a systematic review. Am J Epidemiol. 2014;179(4):423-31. doi: 10.1093/aje/kwt286.

11. Joseph L, Gyorkos TW, Coupal L. Bayesian Estimation of Disease Prevalence and the Parameters of Diagnostic Tests in the Absence of a Gold Standard. Am J Epidemiol. 1995;141(3):263-72. doi: 10.1093/oxfordjournals.aje.a117428.

12. Plummer M, editor JAGS: A program for analysis of Bayesian graphical models using Gibbs sampling. Proceedings of the 3rd international workshop on distributed statistical computing; 2003: Vienna, Austria.

13. Plummer M. rjags: Bayesian Graphical Models using MCMC. 2019.

14. R Core Team. R: A language and environment for statistical computing. R Foundation for Statistical Computing, Vienna, Austria. 2012. 2021.

15. Curtis S. mcmcplots: Create plots from MCMC output. v. 0.4.3 ed2018.

16. Plummer M, Best N, Cowles K, Vines K. CODA: convergence diagnosis and output analysis for MCMC. R news. 2006;6(1):7-11.

17. Brooks SP, Gelman A. General Methods for Monitoring Convergence of Iterative Simulations. J Comput Graph Stat. 1998;7(4):434-55. doi: 10.1080/10618600.1998.10474787.

18. Ntzoufras I. Bayesian modeling using WinBUGS: John Wiley & Sons; 2011.

19. Raftery AE, Lewis S. How many iterations in the Gibbs sampler? Washington Univ Seattle Dept of Statistics, 1991.

20. Toft N, Akerstedt J, Tharaldsen J, Hopp P. Evaluation of three serological tests for diagnosis of Maedi-Visna virus infection using latent class analysis. Vet Microbiol. 2007;120(1-2):77-86. doi: 10.1016/j.vetmic.2006.10.025.

21. Branscum AJ, Gardner IA, Johnson WO. Estimation of diagnostic-test sensitivity and specificity through Bayesian modeling. Prev Vet Med. 2005;68(2-4):145-63. doi: 10.1016/j.prevetmed.2004.12.005.

# Data

| **population** | **ruminant** | **Site** | **Sb** | **Sc** | **Hybrid** | **Hemastix** | **Poc-cca** | **UCCA** | **UCAA** | **MHT** | **KK** | **pgs** | **Direct_diag_abattoir** |
| --- | --- | --- | --- | --- | --- | --- | --- | --- | --- | --- | --- | --- | --- |
| abattoir | bovine | RT |  |  |  | 1 | 0 | 0 | 0 | 0 | 0 | 0 | 0 |
| abattoir | bovine | RT | 1 | 1 | 1 | 1 | 1 | 1 | 1 | 0 | 1 | 1 | 1 |
| abattoir | bovine | RT | 1 | 1 |  | 1 |  | 1 | 1 | 1 | 1 | 1 | 1 |
| abattoir | bovine | BK |  | 1 |  | 1 | 1 | 0 | 1 | 0 | 0 | 1 | 1 |
| abattoir | bovine | BK |  | 1 |  | 1 | 1 | 1 | 1 | 1 | 1 | 1 | 1 |
| abattoir | goat | BK |  |  |  | 0 | 0 | 0 | 1 | 0 | 0 | 1 | 0 |
| abattoir | goat | BK |  |  |  | 0 | 1 | 0 | 1 | 0 | 0 | 1 | 0 |
| abattoir | goat | BK |  | 1 |  | 1 | 0 | 0 | 0 | 0 | 0 | 0 | 1 |
| abattoir | goat | BK |  |  |  | 0 | 0 | 0 | 0 | 0 | 0 | 0 | 0 |
| abattoir | goat | BK |  |  |  | 1 | 1 | 1 | 1 | 0 | 0 | 1 | 0 |
| abattoir | goat | BK |  |  |  | 1 | 0 | 0 | 1 | 0 | 0 | 1 | 0 |
| abattoir | goat | BK |  |  |  | 1 | 0 | 0 | 0 | 0 | 0 | 0 | 0 |
| abattoir | goat | BK |  |  |  | 0 | 0 | 0 | 0 | 0 | 0 | 0 | 0 |
| abattoir | goat | BK |  |  |  | 1 | 0 | 0 | 0 | 0 | 0 | 0 | 0 |
| abattoir | goat | BK |  |  |  | 0 | 1 | 1 | 0 | 0 | 0 | 0 | 0 |
| abattoir | goat | BK |  | 1 |  | 1 | 1 | 0 | 1 | 1 | 0 | 1 | 1 |
| abattoir | goat | BK |  |  |  | 1 | 0 | 0 | 0 | 0 | 0 | 0 | 0 |
| abattoir | goat | BK |  |  |  | 1 | 0 | 0 | 1 | 0 | 0 | 1 | 0 |
| abattoir | sheep | BK |  | 1 |  | 1 | 0 | 0 | 0 | 0 |  | 0 | 1 |
| abattoir | goat | RT |  |  |  | 1 | 0 | 0 | 1 | 0 | 0 | 1 | 0 |
| abattoir | goat | RT |  |  |  | 1 | 1 | 0 | 0 | 0 | 0 | 0 | 0 |
| abattoir | goat | RT |  |  |  | 1 | 0 | 0 | 1 |  | 0 | 1 | 0 |
| abattoir | bovine | RT | 1 |  |  | 0 | 1 | 1 | 1 |  |  | 1 | 1 |
| abattoir | bovine | RT | 1 |  |  | 1 | 1 | 1 | 1 | 0 | 0 | 1 | 1 |
| abattoir | bovine | RT | 1 |  |  | 1 | 1 | 1 | 0 | 0 | 0 | 0 | 1 |
| abattoir | bovine | RT |  |  |  | 1 | 0 | 1 | 1 | 0 | 0 | 1 | 0 |
| abattoir | bovine | BK |  |  |  | 0 | 0 | 0 | 0 | 0 | 1 | 1 | 1 |
| abattoir | bovine | BK |  | 1 |  | 0 | 1 | 1 | 1 | 1 | 1 | 1 | 1 |
| abattoir | bovine | BK |  | 1 | 1 | 1 | 0 | 0 | 1 | 0 | 0 | 1 | 1 |
| abattoir | bovine | BK |  |  |  | 0 | 0 | 0 | 0 | 0 | 0 | 0 | 0 |
| abattoir | bovine | BK |  | 1 |  | 1 | 1 | 1 | 1 | 0 | 1 | 1 | 1 |
| abattoir | bovine | BK |  | 1 |  | 1 | 1 | 0 | 1 |  |  | 1 | 1 |
| abattoir | bovine | BK | 1 | 1 | 1 |  |  | 1 | 1 | 1 | 0 | 1 | 1 |
| abattoir | bovine | BK |  |  |  | 1 | 1 | 0 | 0 | 0 | 0 | 0 | 0 |
| abattoir | bovine | BK |  | 1 | 1 | 1 | 1 | 0 | 1 | 0 | 0 | 1 | 1 |
| abattoir | bovine | BK |  | 1 | 1 | 1 | 1 | 0 | 1 | 1 | 1 | 1 | 1 |
| abattoir | bovine | BK | 1 | 1 | 1 | 1 | 1 | 1 | 1 | 1 | 1 | 1 | 1 |
| abattoir | goat | BK |  |  |  | 0 | 0 | 0 | 0 | 0 | 0 | 0 | 0 |
| abattoir | goat | BK |  |  |  | 0 | 0 | 0 | 0 | 0 | 0 | 0 | 0 |
| abattoir | goat | BK |  |  |  | 1 | 0 | 0 | 1 | 0 | 0 | 1 | 0 |
| abattoir | goat | BK |  |  |  | 0 | 0 | 0 | 0 | 0 | 0 | 0 | 0 |
| abattoir | goat | BK |  |  |  | 1 | 0 | 0 | 0 | 0 | 0 | 0 | 0 |
| abattoir | goat | BK |  |  |  | 1 | 1 | 1 | 1 | 1 | 0 | 1 | 1 |
| abattoir | sheep | BK |  |  |  | 1 | 0 | 0 | 0 | 0 | 0 | 0 | 0 |
| abattoir | sheep | BK |  |  |  | 0 | 0 | 0 | 1 | 0 | 0 | 1 | 0 |
| abattoir | goat | RT |  |  |  | 1 | 1 | 1 | 1 | 0 | 0 | 1 | 0 |
| abattoir | goat | RT |  |  |  | 1 | 0 | 0 | 1 | 0 | 0 | 1 | 0 |
| abattoir | goat | RT |  |  |  | 0 | 0 | 0 | 1 | 0 | 0 | 1 | 0 |
| abattoir | goat | RT |  |  |  | 1 | 1 | 1 | 1 | 0 | 0 | 1 | 0 |
| abattoir | goat | RT |  |  |  | 0 | 0 | 0 | 0 | 0 | 0 | 0 | 0 |
| abattoir | bovine | RT |  |  |  | 0 | 0 | 0 | 0 | 0 | 0 | 0 | 0 |
| abattoir | bovine | RT | 1 |  |  | 1 | 1 | 1 | 1 | 1 | 1 | 1 | 1 |
| abattoir | bovine | RT |  |  |  | 1 | 1 | 1 | 0 | 0 | 1 | 1 | 1 |
| abattoir | bovine | RT |  |  |  | 1 | 1 | 1 | 1 | 0 | 0 | 1 | 0 |
| abattoir | bovine | RT | 1 |  |  | 1 | 0 | 0 | 1 | 1 | 0 | 1 | 1 |
| abattoir | bovine | RT | 1 |  |  | 1 | 0 | 0 | 1 | 1 | 0 | 1 | 1 |
| abattoir | bovine | RT | 1 |  |  | 0 | 1 | 0 | 1 | 1 | 0 | 1 | 1 |
| abattoir | bovine | RT |  |  |  | 1 | 0 | 0 | 1 | 0 | 0 | 1 | 0 |
| abattoir | bovine | RT | 1 |  |  | 1 | 0 | 0 | 1 | 1 | 1 | 1 | 1 |
| abattoir | bovine | RT |  |  |  | 0 | 1 | 1 | 1 | 0 | 1 | 1 | 1 |
| abattoir | bovine | RT | 1 |  |  | 1 | 1 | 1 | 1 | 1 | 1 | 1 | 1 |
| abattoir | bovine | RT | 1 |  |  | 1 | 1 | 1 | 1 | 1 | 1 | 1 | 1 |
| abattoir | bovine | RT | 1 |  |  | 1 | 0 | 0 | 1 | 1 | 1 | 1 | 1 |
| abattoir | bovine | RT |  |  |  | 0 | 1 | 1 | 1 | 0 | 1 | 1 | 1 |
| abattoir | bovine | RT | 1 |  |  | 1 | 1 | 1 | 1 | 1 | 0 | 1 | 1 |
| abattoir | bovine | RT |  |  |  | 1 | 0 | 0 | 0 | 0 | 0 | 0 | 0 |
| abattoir | bovine | RT | 1 |  |  | 1 | 1 | 1 | 1 | 1 | 0 | 1 | 1 |
| abattoir | bovine | RT | 1 |  |  | 1 | 0 | 0 | 1 | 1 | 0 | 1 | 1 |
| abattoir | bovine | RT |  | 1 |  | 1 | 0 | 0 | 1 | 0 | 0 | 1 | 1 |
| abattoir | bovine | RT |  |  |  | 0 | 1 | 1 | 0 | 0 | 0 | 0 | 0 |
| abattoir | goat | RT |  |  |  | 1 | 0 | 0 | 0 | 0 | 0 | 0 | 0 |
| abattoir | goat | RT |  |  |  | 1 | 0 | 0 | 0 | 0 | 0 | 0 | 0 |
| abattoir | goat | RT |  |  |  | 1 | 0 | 0 | 1 | 0 | 0 | 1 | 0 |
| abattoir | goat | RT |  |  |  | 0 | 0 | 0 | 0 | 0 | 0 | 0 | 0 |
| abattoir | goat | RT |  |  |  | 0 | 0 | 1 | 0 | 0 | 0 | 0 | 0 |
| abattoir | goat | RT |  |  |  | 1 | 0 | 0 | 1 | 0 | 1 | 1 | 1 |
| abattoir | goat | RT |  |  |  | 1 | 0 | 0 | 1 | 0 | 0 | 1 | 0 |
| abattoir | goat | RT |  |  |  | 1 | 0 | 0 | 1 | 0 |  | 1 | 0 |
| abattoir | goat | RT |  |  |  | 1 | 0 | 0 | 1 | 0 | 0 | 1 | 0 |
| abattoir | goat | RT |  |  |  | 0 | 0 | 0 | 0 | 0 | 0 | 0 | 0 |
| abattoir | goat | RT | 1 |  |  | 1 | 1 | 1 | 0 | 0 | 0 | 0 | 1 |
| abattoir | goat | RT | 1 |  |  | 1 | 0 | 0 | 0 | 1 | 0 | 1 | 1 |
| abattoir | goat | RT |  |  |  | 1 | 0 | 1 | 0 | 0 | 0 | 0 | 0 |
| abattoir | sheep | RT |  |  |  | 0 | 0 | 0 | 1 | 0 | 0 | 1 | 0 |
| abattoir | bovine | RT |  | 1 | 1 | 1 | 0 | 0 | 1 | 1 | 0 | 1 | 1 |
| abattoir | goat | RT |  |  |  | 1 | 0 | 0 | 1 | 0 | 1 | 1 | 1 |
| abattoir | goat | RT |  |  |  | 0 | 0 | 0 | 1 | 0 | 0 | 1 | 0 |
| abattoir | goat | RT |  |  |  | 1 | 0 | 0 | 1 | 0 | 0 | 1 | 0 |
| abattoir | goat | RT |  |  |  | 1 | 0 | 0 | 1 | 0 | 0 | 1 | 0 |
| live | bovine | BK |  |  |  | 0 | 0 | 0 | 1 | 0 | 0 | 1 |  |
| live | bovine | BK |  |  |  | 0 | 1 | 1 | 1 | 0 | 0 | 1 |  |
| live | bovine | BK |  |  |  |  |  | 0 | 1 | 0 | 0 | 1 |  |
| live | bovine | BK |  |  |  | 0 | 1 | 1 | 1 |  | 0 | 1 |  |
| live | bovine | BK |  |  |  |  |  | 1 | 1 |  | 0 | 1 |  |
| live | bovine | BK |  |  |  | 0 | 1 | 1 | 1 |  | 0 | 1 |  |
| live | goat | BK |  |  |  | 0 | 0 | 0 | 1 |  | 0 | 1 |  |
| live | goat | BK |  |  |  | 0 | 0 | 0 | 1 | 0 | 0 | 1 |  |
| live | sheep | BK |  |  |  | 0 | 0 | 1 | 0 | 0 | 0 | 0 |  |
| live | sheep | BK |  |  |  | 0 | 1 | 0 | 0 | 0 | 0 | 0 |  |
| live | sheep | BK |  |  |  | 0 | 0 | 0 | 1 |  | 0 | 1 |  |
| live | sheep | BK |  |  |  | 0 | 0 | 1 | 1 |  | 0 | 1 |  |
| live | sheep | BK |  |  |  | 0 | 1 | 1 | 1 | 0 | 0 | 1 |  |
| live | sheep | BK |  |  |  | 0 | 0 | 0 | 1 |  | 0 | 1 |  |
| live | sheep | BK |  |  |  | 0 | 0 | 0 | 1 |  | 0 | 1 |  |
| live | sheep | BK |  |  |  |  |  | 1 | 1 | 0 | 0 | 1 |  |
| live | sheep | BK |  |  |  | 0 | 0 | 1 | 1 |  | 0 | 1 |  |
| live | sheep | BK |  |  |  | 0 | 0 | 1 | 1 | 0 | 0 | 1 |  |
| live | sheep | BK |  | 1 |  | 0 | 1 | 1 | 1 | 1 | 1 | 1 |  |
| live | sheep | BK |  |  |  | 0 | 1 | 1 | 1 |  | 0 | 1 |  |
| live | sheep | BK |  |  |  | 0 | 1 | 1 | 0 | 0 | 0 | 0 |  |
| live | sheep | BK |  |  |  | 0 | 0 | 0 | 0 | 0 | 0 | 0 |  |
| live | sheep | BK |  |  |  | 0 | 0 | 0 | 0 |  | 0 | 0 |  |
| live | sheep | BK |  | 1 |  | 0 | 0 | 1 | 0 | 1 | 0 | 1 |  |
| live | sheep | BK |  |  |  | 0 | 0 | 1 | 1 |  | 0 | 1 |  |
| live | sheep | BK |  |  |  | 0 | 0 | 0 | 0 | 0 | 0 | 0 |  |
| live | sheep | BK |  |  |  | 0 | 1 | 1 | 0 |  |  | 0 |  |
| live | sheep | BK |  |  |  | 0 | 1 | 1 | 1 |  | 0 | 1 |  |
| live | sheep | BK |  |  |  | 0 | 0 | 0 | 0 | 0 | 0 | 0 |  |
| live | sheep | BK |  |  |  | 0 | 0 | 0 | 0 | 0 | 0 | 0 |  |
| live | sheep | BK |  |  |  | 0 | 1 | 1 | 1 |  | 0 | 1 |  |
| live | sheep | BK |  |  |  | 0 | 0 | 0 | 0 | 0 | 0 | 0 |  |
| live | sheep | BK |  |  |  | 0 | 0 | 1 | 0 | 0 | 0 | 0 |  |
| live | sheep | BK |  |  |  | 0 | 0 | 0 | 0 | 0 | 0 | 0 |  |
| live | sheep | BK |  |  |  | 0 | 0 | 0 | 0 | 0 | 0 | 0 |  |
| live | sheep | BK |  |  |  | 0 | 1 | 1 | 1 |  | 0 | 1 |  |
| live | bovine | RT | 1 |  |  | 0 | 1 | 0 | 1 | 1 | 0 | 1 |  |
| live | bovine | RT | 1 |  |  | 0 | 0 | 0 | 0 | 1 | 0 | 1 |  |
| live | bovine | RT | 1 |  |  | 0 | 0 | 0 | 1 | 1 | 0 | 1 |  |
| live | goat | RT |  |  |  | 0 | 0 | 1 | 1 | 0 | 0 | 1 |  |
| live | goat | RT | 1 |  |  | 0 | 0 | 0 | 1 | 1 | 0 | 1 |  |
| live | sheep | RT |  |  |  | 0 | 0 | 0 | 1 | 0 | 0 | 1 |  |
| live | sheep | RT |  |  |  | 0 | 0 | 1 | 0 | 0 | 0 | 0 |  |
| live | sheep | RT |  |  |  | 0 | 0 | 0 | 0 | 0 | 0 | 0 |  |
| live | sheep | RT |  |  |  | 0 | 0 | 0 | 1 | 0 | 0 | 1 |  |
| live | sheep | RT |  |  |  | 0 | 0 | 0 | 1 | 0 | 0 | 1 |  |
| live | sheep | RT |  |  |  | 0 | 1 | 1 | 1 | 0 | 0 | 1 |  |
| live | sheep | RT | 1 |  |  | 0 | 0 | 0 | 1 | 1 | 0 | 1 |  |
| live | sheep | RT |  |  |  | 0 | 0 | 0 | 0 | 0 | 0 | 0 |  |
| live | sheep | RT |  |  |  | 0 | 0 | 0 | 0 | 0 | 0 | 0 |  |
| live | sheep | RT |  |  |  | 0 | 0 | 0 | 0 | 0 | 0 | 0 |  |
| live | bovine | RT |  |  |  | 0 | 0 | 0 | 0 | 0 | 0 | 0 |  |
| live | sheep | RT |  |  |  | 0 | 0 | 1 | 1 | 0 | 0 | 1 |  |
| live | sheep | RT |  |  |  | 0 | 0 | 0 | 1 | 0 | 0 | 1 |  |
| live | sheep | BK |  |  |  | 0 | 0 | 0 | 0 |  | 0 | 0 |  |
| live | sheep | RT |  |  |  | 0 | 0 | 0 | 1 | 0 | 0 | 1 |  |
| live | sheep | BK |  |  |  | 0 | 0 | 0 | 1 |  | 0 | 1 |  |
| live | sheep | BK |  |  |  | 0 | 0 | 1 | 1 | 0 | 0 | 1 |  |
| live | sheep | RT |  |  |  | 0 | 0 | 0 | 1 | 0 | 0 | 1 |  |
| live | sheep | RT |  |  |  | 0 | 0 | 0 | 0 | 0 | 0 | 0 |  |
| live | sheep | BK |  |  |  | 0 | 1 | 0 | 0 |  | 0 | 0 |  |
| live | sheep | RT |  |  |  | 0 | 0 | 0 | 0 | 0 | 0 | 0 |  |
| live | sheep | BK |  |  |  | 0 | 0 | 1 | 0 |  | 0 | 0 |  |
| live | bovine | BK |  |  |  | 0 | 1 | 1 | 1 | 0 | 0 | 1 |  |
| live | bovine | BK |  |  |  | 0 | 1 | 1 | 1 |  | 0 | 1 |  |
| live | goat | BK |  |  |  | 0 | 0 | 1 | 1 | 0 | 0 | 1 |  |
| live | goat | BK |  |  |  | 0 | 0 | 0 | 1 | 0 | 0 | 1 |  |
| live | goat | BK |  |  |  | 0 | 0 | 1 | 0 | 0 | 0 | 0 |  |
| live | goat | BK |  |  |  | 0 | 1 | 1 | 0 | 0 | 0 | 0 |  |
| live | goat | BK |  | 1 |  | 0 | 0 | 1 | 0 | 1 | 0 | 1 |  |
| live | goat | BK |  |  |  | 0 | 0 | 1 | 1 | 0 | 1 | 1 |  |
| live | goat | BK |  |  |  | 0 | 0 | 1 | 1 | 0 |  | 1 |  |
| live | goat | BK |  |  |  | 0 | 1 | 1 | 1 | 0 | 0 | 1 |  |
| live | goat | BK |  |  |  | 0 | 0 | 1 | 0 | 0 | 0 | 0 |  |
| live | sheep | BK |  |  |  | 0 | 0 | 0 | 0 | 0 | 0 | 0 |  |
| live | sheep | BK |  |  |  | 0 | 0 | 1 | 1 | 0 | 0 | 1 |  |
| live | sheep | BK |  |  |  | 0 | 1 | 1 | 1 | 0 | 0 | 1 |  |
| live | sheep | BK |  |  |  | 0 | 0 | 0 | 0 | 0 | 0 | 0 |  |
| live | sheep | BK |  |  |  | 0 | 0 | 0 | 1 | 0 | 1 | 1 |  |
| live | sheep | BK |  |  |  | 0 | 0 | 0 | 0 |  | 0 | 0 |  |
| live | sheep | BK |  |  |  | 0 | 1 | 0 | 1 | 1 | 0 | 1 |  |
| live | sheep | BK |  |  |  | 0 | 1 | 1 | 1 | 0 | 0 | 1 |  |
| live | sheep | BK |  |  |  | 0 | 0 | 1 | 1 |  | 0 | 1 |  |
| live | sheep | BK |  |  |  | 0 | 1 | 1 | 1 | 0 | 0 | 1 |  |
| live | sheep | BK |  |  |  | 0 | 0 | 0 | 0 |  | 0 | 0 |  |
| live | sheep | BK |  |  |  | 0 | 0 | 0 | 0 |  | 0 | 0 |  |
| live | sheep | BK |  |  |  | 0 | 0 | 1 | 1 | 0 | 0 | 1 |  |
| live | sheep | BK |  | 1 |  | 0 | 1 | 1 | 1 | 1 | 1 | 1 |  |
| live | sheep | BK |  |  |  | 0 | 0 | 0 | 0 |  | 0 | 0 |  |
| live | sheep | BK |  |  |  | 0 | 0 | 0 | 0 | 0 | 0 | 0 |  |
| live | sheep | BK |  |  |  | 0 | 1 | 1 | 1 | 0 | 0 | 1 |  |
| live | sheep | BK |  |  |  | 0 | 0 | 0 | 1 | 0 | 0 | 1 |  |
| live | sheep | BK |  |  |  | 0 | 0 | 0 | 0 | 0 | 0 | 0 |  |
| live | sheep | BK |  |  |  | 0 | 1 | 0 | 0 | 0 | 1 | 1 |  |
| live | sheep | BK |  |  |  | 0 | 0 | 1 | 0 | 0 | 0 | 0 |  |
| live | sheep | BK |  |  |  | 1 | 1 | 0 | 1 | 0 | 0 | 1 |  |
| live | sheep | BK |  |  |  | 0 | 0 | 0 | 1 | 0 | 0 | 1 |  |
| live | sheep | BK |  |  |  | 0 | 0 | 0 | 0 | 0 | 1 | 1 |  |
| live | sheep | RT |  |  |  | 0 | 0 | 0 | 0 | 0 | 0 | 0 |  |
| live | bovine | RT | 1 |  |  | 0 | 1 | 1 | 1 | 1 | 0 | 1 |  |
| live | bovine | RT | 1 |  |  | 0 | 1 | 1 | 1 | 1 | 1 | 1 |  |
| live | bovine | RT | 1 |  |  | 0 | 1 | 0 | 1 | 1 | 1 | 1 |  |
| live | sheep | RT |  |  |  | 0 | 0 | 1 | 0 | 0 | 0 | 0 |  |
| live | sheep | RT |  |  |  | 0 | 0 | 0 | 0 | 0 | 0 | 0 |  |
| live | sheep | RT |  |  |  | 0 | 0 | 0 | 0 | 0 | 0 | 0 |  |
| live | sheep | RT |  |  |  | 0 | 0 | 0 | 1 |  |  | 1 |  |
